# Comparison of Various Nuclear Localization Signal-Fused Cas9 Proteins and *Cas9* mRNA for Genome Editing in Zebrafish

**DOI:** 10.1534/g3.117.300359

**Published:** 2018-01-02

**Authors:** Peinan Hu, Xueying Zhao, Qinghua Zhang, Weiming Li, Yao Zu

**Affiliations:** * International Research Center for Marine Biosciences, Ministry of Science and Technology, College of Fisheries and Life Sciences, Shanghai Ocean University, 201306, P.R. China; † Key Laboratory of Exploration and Utilization of Aquatic Genetic Resources, Ministry of Education, College of Fisheries and Life Sciences, Shanghai Ocean University, 201306, P.R. China; ‡ National Demonstration Center for Experimental Fisheries Science Education, Shanghai Ocean University, Shanghai 201306, P.R. China; § Department of Fisheries and Wildlife, Michigan State University, East Lansing, Michigan 48824

**Keywords:** CRISPR/Cas9, Cas9 protein, knockout, efficiency, NLS, zebrafish

## Abstract

The clustered regularly interspaced short palindromic repeats (CRISPR)/Cas9 system has been proven to be an efficient and precise genome editing technology in various organisms. However, the gene editing efficiencies of Cas9 proteins with a nuclear localization signal (NLS) fused to different termini and *Cas9* mRNA have not been systematically compared. Here, we compared the ability of Cas9 proteins with NLS fused to the N-, C-, or both the N- and C-termini and N-NLS-Cas9-NLS-C mRNA to target two sites in the *tyr* gene and two sites in the *gol* gene related to pigmentation in zebrafish. Phenotypic analysis revealed that all types of Cas9 led to hypopigmentation in similar proportions of injected embryos. Genome analysis by T7 Endonuclease I (T7E1) assays demonstrated that all types of Cas9 similarly induced mutagenesis in four target sites. Sequencing results further confirmed that a high frequency of indels occurred in the target sites (*tyr1* > 66%, *tyr2* > 73%, *gol1* > 50%, and *gol2* > 35%), as well as various types (more than six) of indel mutations observed in all four types of Cas9-injected embryos. Furthermore, all types of Cas9 showed efficient targeted mutagenesis on multiplex genome editing, resulting in multiple phenotypes simultaneously. Collectively, we conclude that various NLS-fused Cas9 proteins and *Cas9* mRNAs have similar genome editing efficiencies on targeting single or multiple genes, suggesting that the efficiency of CRISPR/Cas9 genome editing is highly dependent on guide RNAs (gRNAs) and gene loci. These findings may help to simplify the selection of Cas9 for gene editing using the CRISPR/Cas9 system.

The type II CRISPR associated with Cas9 endonuclease (CRISPR/Cas9) system has emerged as a powerful tool for studying gene function (Jinek *et al.* 2012). The CRISPR/Cas9 system has a high genome editing efficiency, is simple to design, is not time-consuming, and has been successfully applied in a variety of model organisms including the mouse (*Mus musculus*) (Shen *et al.* 2013), fruit fly (*Drosophila melanogaster*) (Bassett *et al.* 2013), zebrafish (*Danio rerio*) (Chang *et al.* 2013; Hwang *et al.* 2013), nematode (*Caenorhabditis elegans*) (Tzur *et al.* 2013), and *Arabidopsis* (*Arabidopsis thaliana*) (Fauser *et al.* 2014).

In the 5 yr since the CRISPR/Cas9 system emerged as a versatile genome editing technology, many researchers have demonstrated that co-injection of *in vitro*-transcribed capped polyadenylated *Cas9* mRNA and gRNA specific to the target genomic locus induces DNA double-strand breaks (DSBs), leading to targeted gene disruption. However, the efficiency of genome editing varies. For instance, Li *et al.* (2014) designed three gRNAs targeting the fifth exon of the mouse Fumarylacetoacetate hydrolase *(Fah)* gene and achieved genomic disruption efficiencies of 54.26, 23.15, and 42.05% for gRNA1, 2, and 3, respectively. In zebrafish, Jao *et al.* (2013) established a highly efficient CRISPR mutagenesis system by modifying the 3′ end of the gRNA with GGAUC, which led to mutation rates >80%.

Injection of Cas9 protein and gRNA can also efficiently induce DSBs. Wu *et al.* (2017) showed that Cas9 protein/sgRNA resulted in a mutation rate of 30–60% during excision of a point mutation in exon 80 to restore correct localization of collagen VII protein in the mouse. Sung *et al.* (2014) found C-NLS-Cas9 protein and *Cas9* mRNA induced similar gene modification efficiency in zebrafish founder embryos. Consistent with these findings, Gagnon *et al.* (2014) reported that C-NLS-Cas9 protein and *Cas9* mRNA both resulted in a high genome editing frequency at calcium/calmodulin-dependent protein kinase (CaM kinase) II γ 1 (*camk2g1*) and connective tissue growth factor a (*ctgfa*) gene target sites in zebrafish, though Cas9 protein induced a higher mutagenesis rate than *Cas9* mRNA at the glutamate receptor, ionotropic, AMPA 3a (*gria3a*), and lysine (K)-specific demethylase 6A, like *utx1* loci. Although these initial pioneering results are encouraging, there has been no comprehensive comparison of the gene editing efficiency of Cas9 proteins and *Cas9* mRNA.

The CRISPR/Cas9 system is based on a prokaryotic immune mechanism that defends against invasion of nucleic acids (Brouns *et al.* 2008). As Cas9 is derived from *Streptococcus pyogenes*, it may be necessary to an attach an NLS so Cas9 can be transported into the nucleus to enable editing of the eukaryotic genome. Cong *et al.* (2013) found that attaching an NLS to both termini of Cas9 protein improved the efficiency of its transport into the nucleus. Mali *et al.* (2013) reported that only adding an NLS to the C-terminus of Cas9 protein was required to maintain the gene editing efficiency of the CRISPR/Cas9 system in eukaryotic cells. Moreover, Shen *et al.* (2013) showed that adding a 32 amino acid linker between the N-terminal NLS and Cas9 protein could improve Cas9 cleavage activity in eukaryotes. However, the gene editing efficiency of Cas9 protein or mRNA with an NLS fused to different termini have not been systematically compared.

In this study, we used four Cas9 proteins: N-NLS-Cas9 protein (called N-Cas9 protein), Cas9-NLS-C protein (C-Cas9 protein), N-NLS-Cas9-NLS-C protein (N-Cas9-C protein), Cas9 protein without NLS (Cas9 protein) and N-NLS-Cas9-NLS-C mRNA (N-Cas9-C mRNA). We targeted two genes related to body pigmentation to make it easy to visually compare gene editing efficiency by assessing pigmentation defects with two sites of each gene. Each one of the two target sites was designed as reported previously (Jao *et al.* 2013), and another one was located in the second exon of *tyrosinase* (*tyr*) gene and the sixth exon of *golden (gol or slc24a5)*. The Cas9 proteins or *Cas9* mRNA were co-injected with synthesized gRNA into single-cell zebrafish embryos to compare gene editing efficiency for two sites in the *tyr* gene and two sites in the *gol* gene. Distinct phenotypes were observed, including hypopigmentation of skin melanophores and the retinal pigmented epithelium in injected *tyr* F_0_ embryos, and hypopigmentation of the retinal pigmented epithelium in injected *gol* F_0_ embryos. Furthermore, we observed that various NLS-fused Cas9 proteins and Cas9 mRNA showed efficient effects on multiplex genome editing in zebrafish. We conclude that the varied NLS-fused Cas9 proteins and *Cas9* mRNA tested in this study lead to similarly efficient gene editing efficiencies.

## Materials and Methods

### Zebrafish husbandry

Zebrafish were maintained in a recirculating system at 28.5° and pH of 7.0–8.0. The circulating water was UV light-treated and aerated. Embryos were collected in purified water and incubated at 28.5°. All handling of fishes was carried out in accordance with the guidelines on the care and use of animals for scientific purposes set up by the Institutional Animal Care and Use Committee (IACUC) of the Shanghai Ocean University (SHOU), Shanghai, China. This research was approved by the IACUC (IACUC 20171009) of SHOU.

### Cas9 plasmid and Cas9 proteins

The full-length codon-optimized Cas9 plasmid was obtained from Bo Zhang’s laboratory (Liu *et al.* 2014). This Cas9 plasmid contains a consensus Kozak sequence at the translational start site, an NLS called a monopartite NLS at the N-terminus whose sequence is PKKKRKV from the SV40 Large T-antigen, and an NLS called a nucleoplasmin NLS at the C-terminus whose sequence is KR[PAATKKAGQA]KKKK. The three Cas9 proteins (N-NLS-Cas9, Cas9-NLS-C, and N-NLS-Cas9-NLS-C) containing the same monopartite NLS, and another Cas9 without an NLS, were obtained from GenScript (Nanjing, China).

### RNA synthesis

The codon-optimized Cas9 plasmid was linearized using *Xba*I and purified using DNA Clean & Concentrator-5 (Zymo Research, Irvine, CA). Capped *Cas9* mRNA was synthesized *in vitro* using the mMESSAGE mMACHINE T7 ULTRA kit (Ambion, New York, NY) as previously described (Liu *et al.* 2014) and purified using the mirVana miRNA Isolation Kit (Ambion).

To make gRNA, double-stranded DNA was amplified by PCR (TransGen Biotech, Beijing, China) from gRNA using the target site-specific forward primer (5′-TAATACGACTCACTATANNNNNNNNNNNNNNNNNNNNGTTTTAGAGCTAGAAATAGC-3′) and the common reverse primer (5′-AAAAAAAGCACCGACTCGGTGCCAC-3′) (Chang *et al.* 2013), then gRNA was generated *in vitro* using the MAXIscript T7 kit (Ambion) and purified using the mirVana miRNA Isolation Kit (Ambion). Each target sequence contains GG at the beginning and NGG as the PAM sequence.

### Microinjection

For microinjection 1 nl of a mixture of gRNA (100 pg) and Cas9 protein (800 pg) or *Cas9* mRNA (400 pg) was directly co-injected into each single-celled embryo [derived from the wild-type (WT) AB incross], as previously described (Tong *et al.* 2014). Approximately 300 embryos were injected every experiment. Each experiment was repeated independently three times.

### Assessment of mutation efficiency

Two days after microinjection of gRNA and Cas9, genomic DNA was extracted from the four groups of pooled microinjected embryos; five injected embryos were randomly selected for each group in each of the three independent experiments. Genomic DNA was used as template and amplified by PCR using the primer pairs shown in Supplemental Material, Table S1 in File S1. The PCR products were denatured and annealed to enable heteroduplex formation, then treated with T7EI (New England Biolabs, Ipswich, MA) for 45 min at 37°. Mutation efficiency was calculated as previously described (Tong *et al.* 2014). The digested PCR products were examined by agarose gel (2%) electrophoresis. The remainder of the PCR products were subjected to Sanger sequencing by Sangon Biotech (Shanghai, China). At each target site, PCR products mixed from three independent experiments were ligated into the pMD19-T TA cloning vector (Takara) and transformed into DH5α competent cells (Tiangen) independently. Approximately 20 individual colonies were sequenced for each amplicon as previously described (Zu *et al.* 2016).

### Statistical analysis

Data derived from three independent experiments were evaluated by one-way ANOVA for comparisons between groups using GraphPad Prism 5. *P* < 0.05 was considered to be statistically significant.

### Data availability

The authors state that all data necessary for confirming the conclusions presented in the article are represented fully within the article.

## Results

### Survival rates of zebrafish injected with various NLS-fused Cas9 proteins and Cas9 mRNA

To investigate the best concentration of Cas9 protein to inject for gene editing, we co-injected 100 pg *gol* target2 gRNA with 400, 600, and 800 pg of N-Cas9 protein, respectively, into one-cell-stage zebrafish embryos. T7E1 assays demonstrated that the 800 pg Cas9 protein had the highest mutagenesis rate (*P* < 0.05), and the proportions of mosaic and null-like phenotypes were also higher in the 800 pg Cas9 group (Figure S1 in File S1). Therefore, we chose 800 pg as the concentration of Cas9 protein to perform the following microinjection experiments.

**Figure 1:**
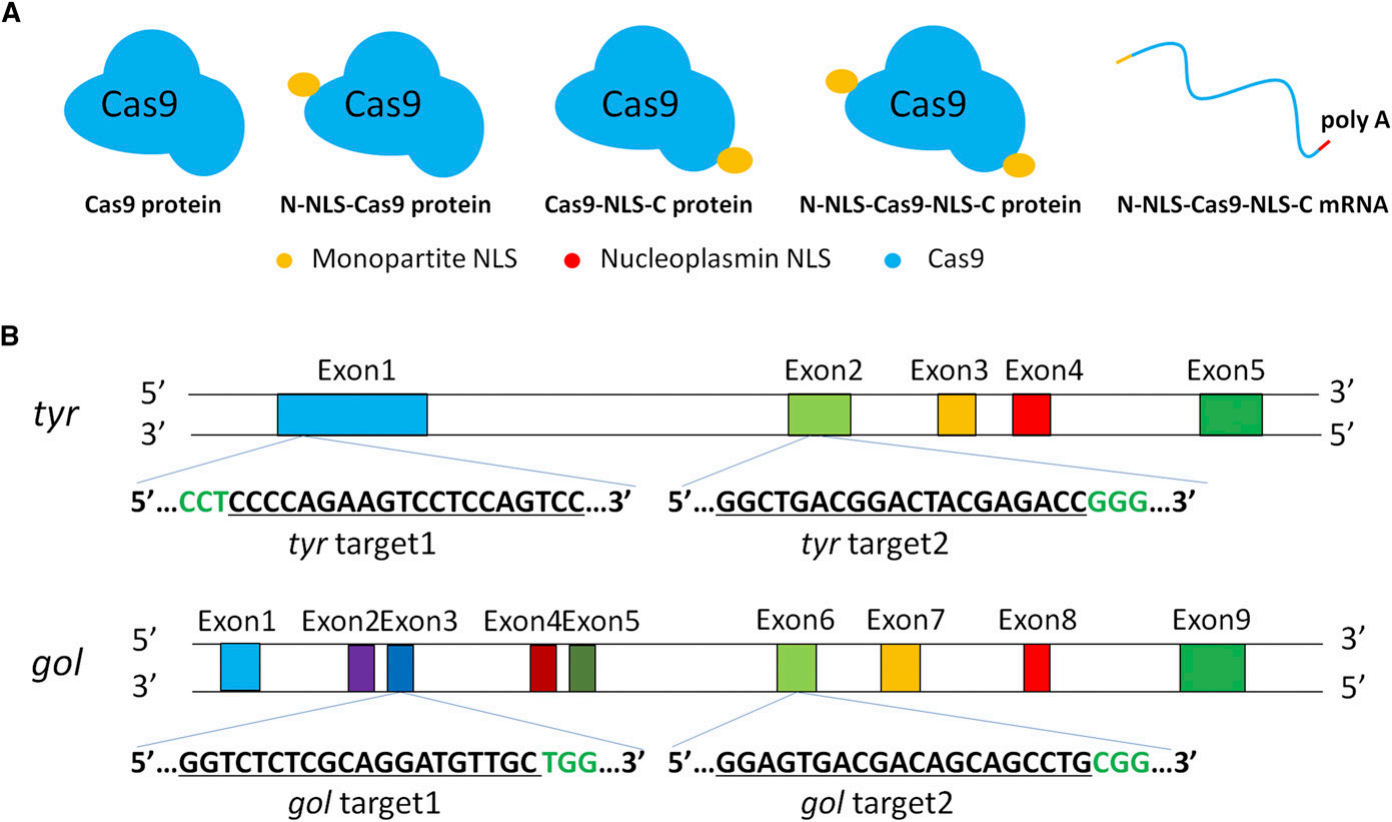
Clustered regularly interspaced short palindromic repeats (CRISPR)/Cas9-mediated gene editing in zebrafish. (A) Schematic illustrating the no nuclear localization signal (NLS) Cas9 protein, various NLS-fused Cas9 proteins, and *Cas9* mRNA. (B) *tyr* target 1 and *gol* target 1 were as previously described, *tyr* target 2 was located in the second exon of *tyr*, and *gol* target 2 in the sixth exon of *gol*.

**Figure 2:**
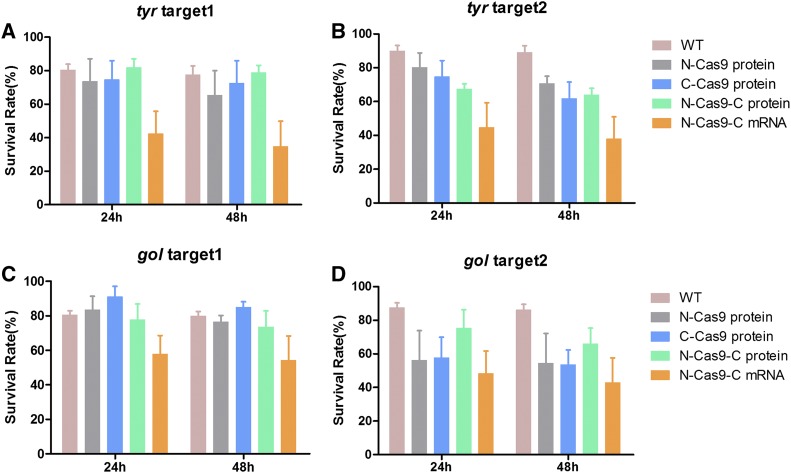
Survival rates of zebrafish embryos after microinjection of various nuclear localization signal (NLS)-fused Cas9 proteins or N-Cas9-C mRNA. Survival rates of zebrafish embryos injected with various NLS-fused Cas9 proteins, or Cas9 mRNA and guide (g)RNA targeting the two target sites in *tyr* (A and B), and two target sites in *gol* (C and D) at 24 and 48 hpf (hr postfertilization). WT, wild-type.

We co-injected 1 nl of 100 pg gRNA and 400 pg N-Cas9-C mRNA, or 800 pg of the three NLS-fused Cas9 proteins (Figure 1A), into single-cell WT zebrafish embryos to target two sites in the *tyr* gene and two sites in the *gol* gene (Figure 1B). For every experiment, ∼300 embryos were injected with specific gRNA and different types of Cas9. The numbers of surviving embryos were counted at 24 and 48 hr postfertilization (hpf). The survival rates at 24 and 48 hpf were similar for each site. Moreover, the survival rates of zebrafish embryos injected with the various NLS-fused Cas9 proteins or N-Cas9-C mRNA were not significantly different (Figure 2, A–D, *P* > 0.05).

### Mutation efficiency of the various NLS-fused Cas9 proteins and Cas9 mRNA for two tyr gene sites

The target sites and target region primers were designed using ZIFIT and NCBI programs (Figure 1B and Table S1 in File S1). The various NLS-fused Cas9 proteins or *Cas9* mRNA were injected into single-cell zebrafish embryos with *tyr* gRNA 1 or gRNA 2. For each target site, embryos were injected with four groups of Cas9 and gRNA in three independent experiments. To evaluate the knockout efficiency of genomic disruption, genomic DNA was extracted from three tubes of pooled microinjected embryos at 48 hpf; each tube contained five randomly selected injected embryos and a pool of five randomly selected uninjected WT embryos as controls. The target fragment was amplified by PCR using genomic DNA as template and the rate of mutagenesis quantified by T7E1 assays and PCR Sanger sequencing.

**Figure 3:**
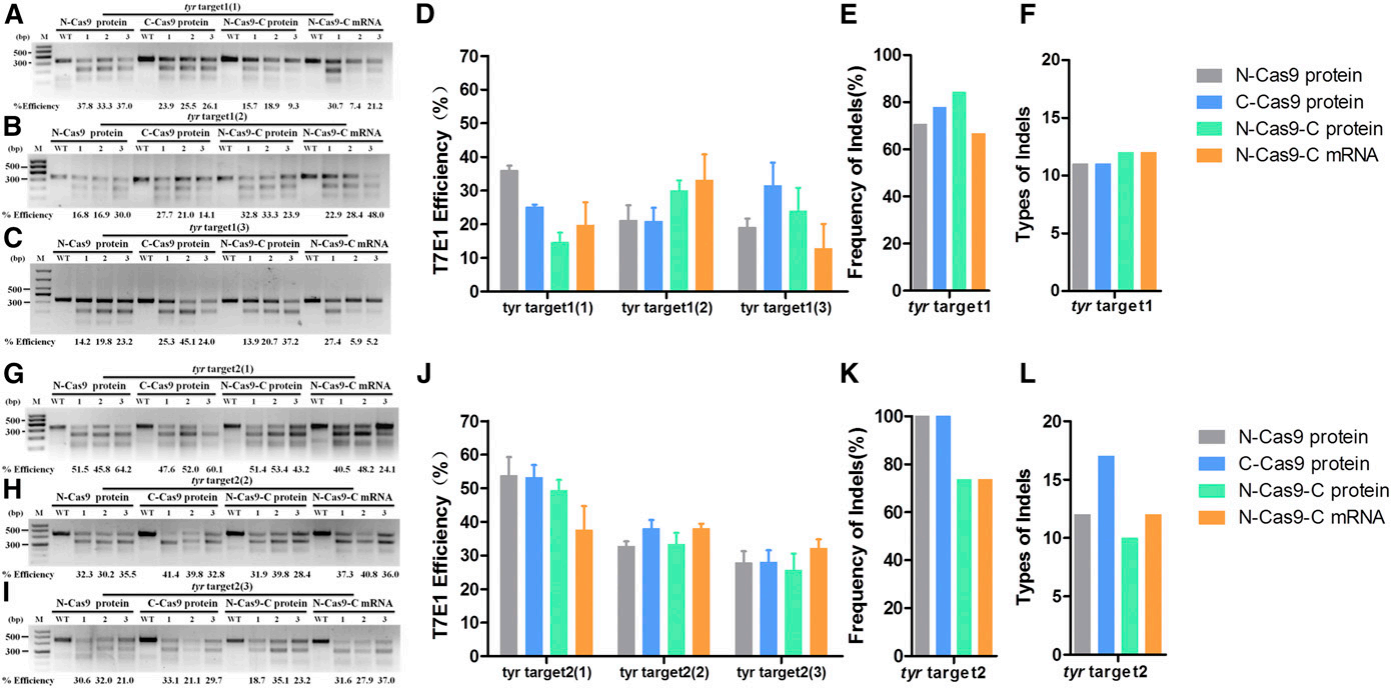
Knockout efficiency of various nuclear localization signal (NLS)-fused Cas9 proteins and N-Cas9-C mRNA for two sites of the *tyr* gene. T7 Endonuclease I (T7E1) assays of the rates of mutagenesis for *tyr* target 1 (A–D) and *tyr* target 2 (G–J) in three tubes of pooled embryos microinjected with relevant guide (g)RNA and the various NLS-fused Cas9 proteins and N-Cas9-C mRNA. Each group contained five randomly selected embryos; three independent experiments were performed for each target. The frequency of indels and types of indel of *tyr* target1 (E and F) and *tyr* target2 (K and L).

For the *tyr* target 1, T7E1 assays showed that the rate of mutagenesis ranged from 14.2 to 37.8% for N-Cas9 protein (first experiment: mean ± SD, 36.03 ± 2.40; second experiment: mean ± SD, 21.23 ± 7.59; and third experiment: mean ± SD, 19.07 ± 4.54) in the triplicate experiments, 14.1–45.1% for C-Cas9 protein (first experiment: mean ± SD, 25.17 ± 1.14; second experiment: mean ± SD, 20.93 ± 6.80; and third experiment: mean ± SD, 31.47 ± 11.82), 9.3–37.2% for N-Cas9-C protein (first experiment: mean ± SD, 14.63 ± 4.89; second experiment: mean ± SD, 30.00 ± 5.29; and third experiment: mean ± SD, 29.93 ± 11.98), and 5.2–48.0% for N-Cas9-C mRNA (first experiment: mean ± SD, 19.77 ± 11.72; second experiment: mean ± SD, 33.10 ± 13.19; and third experiment: mean ± SD, 12.83 ± 12.62) (Figure 3, A–D). The corresponding PCR Sanger sequences are shown in Figure S4 in File S1. To further analyze the different types of indels induced by each Cas9 protein and Cas9 mRNA, we sequenced 20 individual colonies of each group and the indel frequencies of all four groups were >66% (Figure 3E and Figure S5 in File S1). There were 11, 11, 12, and 12 types of indel, respectively, documented in the sequences of the N-Cas9 protein-, C-Cas9 protein-, N-Cas9-C protein-, and N-Cas9-C mRNA-targeted embryos (Figure 3F and Figure S5 in File S1). These results indicated that there was no significant difference in knockout efficiency between the various NLS-fused Cas9 proteins and N-Cas9-C mRNA for *tyr* target 1 (first experiment: *P* = 0.019, and second and third experiments: *P* > 0.05).

T7E1 assays of the *tyr* gRNA2 samples at 48 hpf revealed that the mutagenesis rate was 21.0–64.2% for N-Cas9 protein (first experiment: mean ± SD, 53.83 ± 9.42; second experiment: mean ± SD, 32.67 ± 2.67; and third experiment: mean ± SD, 27.87 ± 5.99), 21.1–60.1% for C-Cas9 protein (first experiment: mean ± SD, 53.23 ± 6.34; second experiment: mean ± SD, 37.93 ± 4.54; and third experiment: mean ± SD, 27.97 ± 6.18), 18.7–53.4% for N-Cas9-C protein (first experiment: mean ± SD, 49.33 ± 5.40; second experiment: mean ± SD, 33.37 ± 5.84; and third experiment: mean ± SD, 25.67 ± 8.47), and 24.1–48.2% for N-Cas9-C mRNA (first experiment: mean ± SD, 37.60 ± 12.31; second experiment: mean ± SD, 38.03 ± 2.48; and third experiment: mean ± SD, 32.17 ± 4.58) (Figure 3, G–J). The corresponding PCR Sanger sequences are shown in Figure S4 in File S1. Besides, we sequenced 20 individual colonies of each group as mentioned above and the indel frequencies of the four groups were >73% (Figure 3K and Figure S6 in File S1). There were 12, 17, 10 and 12 types of indels, respectively, documented in the sequences of the N-Cas9 protein-, C-Cas9 protein-, N-Cas9-C protein- and N-Cas9-C mRNA-injected embryos (Figure 3L and Figure S6 in File S1). The results showed that there was no significant difference in knockout efficiency between the various NLS-fused Cas9 proteins and N-Cas9-C mRNA for *tyr* target 2 (all *P* > 0.05).

**Figure 4:**
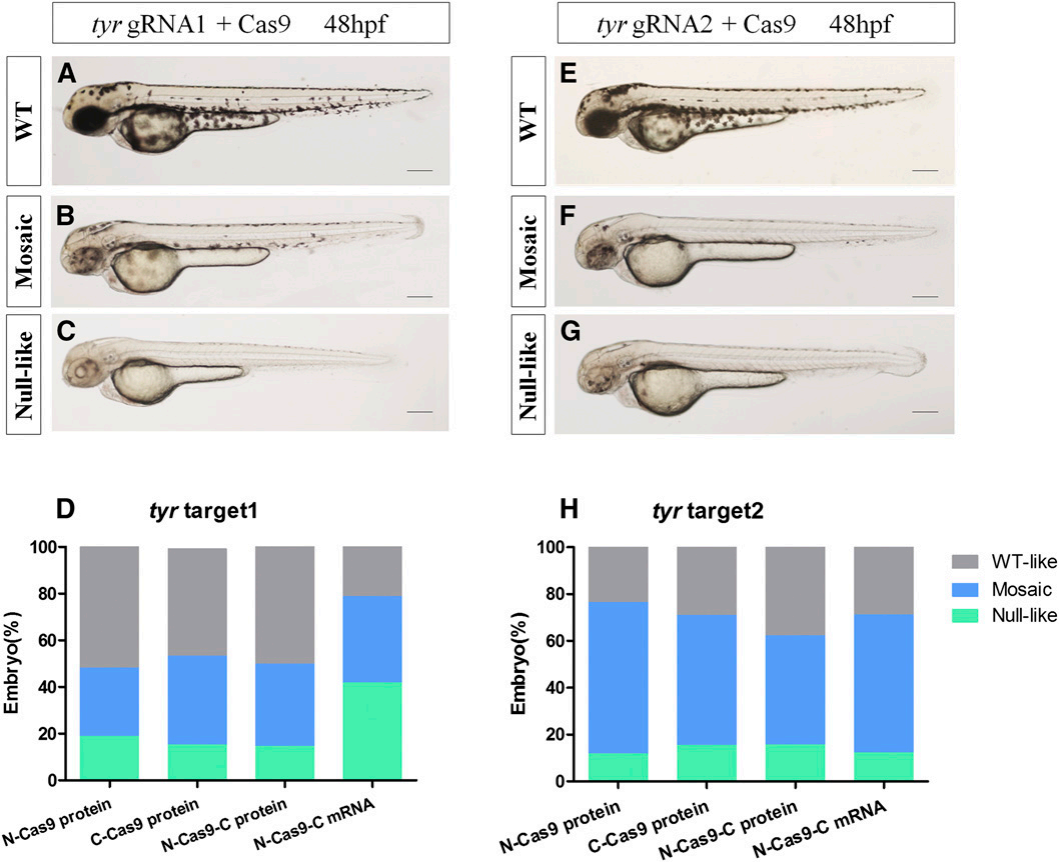
Mutagenetic phenotype of various nuclear localization signal (NLS)-fused Cas9 proteins and N-Cas9-C mRNA for two sites of the *tyr* gene. Lateral views of wild-type (WT) (A and E) and *tyr*-target 1- (B and C) or *tyr*-target 2- injected (F and G) embryos at 48 hpf (hr postfertilization). The phenotypes observed were WT (A and E), mosaic hypopigmentation (B and F), and null-like (C and G). Proportions of embryos with each phenotype (D and H). Scale bars: 0.2 mm.

The *tyr* gene is required for the conversion of tyrosinase into melanin (Page-McCaw *et al.* 2004). Targeting *tyr* resulted in three phenotypes at 48 hpf: WT-like (similar to uninjected embryos), mosaic (hypopigmentation on embryo body), and null-like (no melanin on embryo body; Figure 4, A–C and E–G). With over 200 embryos, we assessed the proportions of embryos with each phenotype at 48 hpf to evaluate phenotypic mutation efficiency and specificity at the phenotype level (Figure 4, D and H). N-Cas9-C mRNA induced a high proportion of the null-like phenotype at *tyr* target 1 (∼40%), while the three Cas9 protein variants induced lower proportions of the null-like phenotype and higher proportions of the WT-like phenotype. N-Cas9-C mRNA and the three Cas9 protein variants led to similar proportions of the mosaic phenotype (Figure 4D). For *tyr* target 2, N-Cas9-C mRNA and the three Cas9 protein variants generated high proportions of the mosaic and null-like phenotype (62.2–76.5%; Figure 4H).

### Mutation efficiency of the various NLS-fused Cas9 proteins and Cas9 mRNA for two gol gene sites

**Figure 5:**
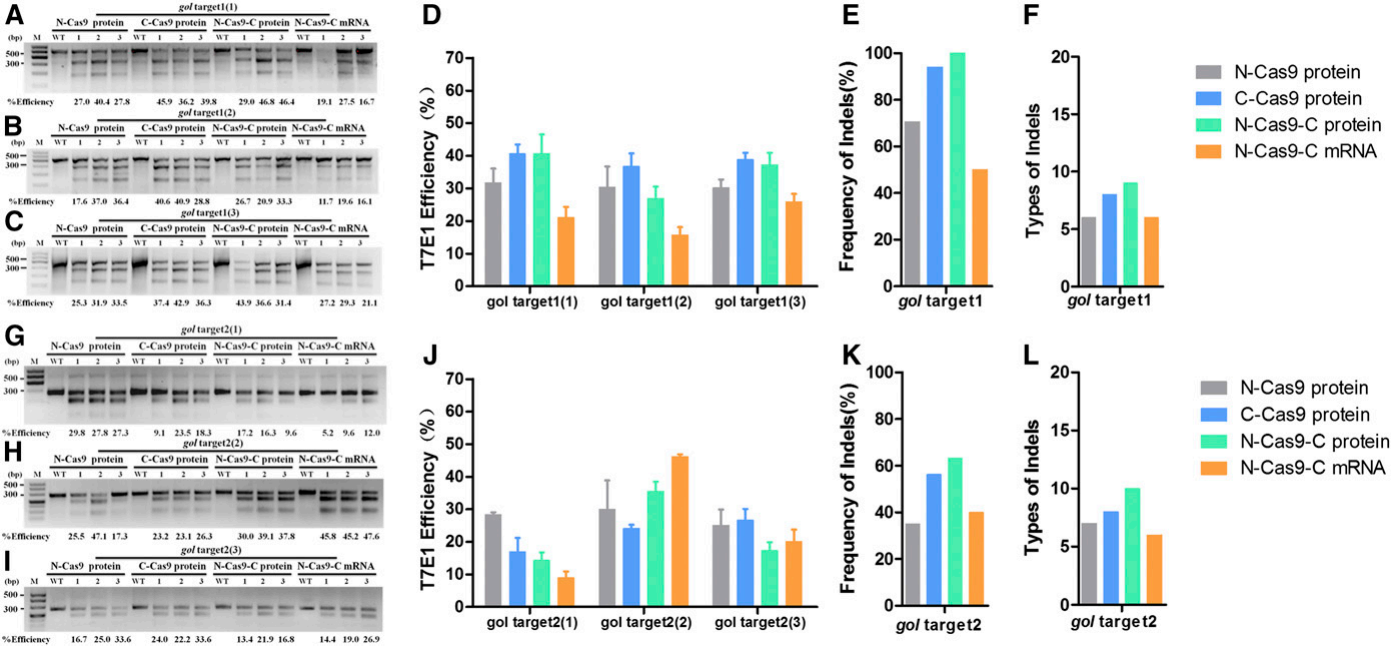
Knockout efficiency of various nuclear localization signal (NLS)-fused Cas9 proteins and N-Cas9-C mRNA for two sites of the *gol* gene. T7 Endonuclease I (T7E1) assays of the rates of mutagenesis for *gol* target 1 (A–D) and gol target 2 (G–J) in three tubes of pooled embryos microinjected with guide (g)RNA and the various NLS-fused Cas9 proteins or N-Cas9-C mRNA. Each group contained five randomly selected embryos; three independent experiments were performed for each target. The frequency of indels and types of indel of *gol* target 1 (E and F) and *gol* target 2 (K and L) are shown.

Next, we evaluated the knockout efficiency of the three Cas9 proteins and *Cas9* mRNA when targeting another pigmentation-related gene, *gol*, which encodes the putative cation exchanger Slc24a5 (solute carrier family 24, member 5) (Lamason *et al.* 2005) (Figure 1B). At *gol* target 1, T7E1 assays showed that N-Cas9 protein induced an genomic disruption rate of 17.6–40.4% (first experiment: mean ± SD, 31.73 ± 7.52; second experiment: mean ± SD, 30.33 ± 11.03; and third experiment: mean ± SD, 30.23 ± 4.35); C-Cas9 protein, 28.8–45.9% (first experiment: mean ± SD, 40.63 ± 4.90; second experiment: mean ± SD, 36.76 ± 6.90; and third experiment: mean ± SD, 38.87 ± 3.54); N-Cas9-C protein, 20.9–46.8% (first experiment: mean ± SD, 40.73 ± 10.16; second experiment: mean ± SD, 26.97 ± 6.20; and third experiment: mean ± SD, 37.30 ± 6.28); and N-Cas9-C mRNA, 11.7–29.3% (first experiment: mean ± SD, 21.10 ± 5.67; second experiment: mean ± SD, 15.80 ± 3.96; and third experiment: mean ± SD, 25.87 ± 4.26) (Figure 5, A–D). The corresponding sequences are shown in Figure S4 in File S1. To further analyze the different types of indels induced by each Cas9 protein and Cas9 mRNA, 20 individual colonies of each group were sequenced. Indels were detected in each group with frequencies >50% (Figure 5E and Figure S7 in File S1), and the types of indels documented in the sequences of the N-Cas9 protein-, C-Cas9 protein-, N-Cas9-C protein-, and N-Cas9-C mRNA-targeted embryos were 7, 6, 9, and 6, respectively (Figure 5F and Figure S7 in File S1). There were no significant differences in knockout efficiency between the three Cas9 protein variants and *Cas9* mRNA for *gol* target 1 (first experiment: *P* = 0.035, and second and third experiments: *P* > 0.05).

For *gol* target 2, the genomic disruption rate ranged from 16.7 to 47.1% for N-Cas9 protein (first experiment: mean ± SD, 28.3 ± 1.32; second experiment: mean ± SD, 29.97 ± 15.39; and third experiment: mean ± SD, 25.10 ± 8.45), 9.1–33.6% for C-Cas9 protein (first experiment: mean ± SD, 16.97 ± 7.29; second experiment: mean ± SD, 24.20 ± 1.82; and third experiment: mean ± SD, 26.60 ± 6.13), 9.6–39.1% for N-Cas9-C protein (first experiment: mean ± SD, 14.37 ± 4.15; second experiment: mean ± SD, 35.63 ± 4.92; and third experiment: mean ± SD, 17.37 ± 4.28), and 5.2–47.6% for N-Cas9-C mRNA (first experiment: mean ± SD, 8.93 ± 3.45; second experiment: mean ± SD, 46.20 ± 1.25; and third experiment: mean ± SD, 20.10 ± 6.32) (Figure 5, G–J). The corresponding sequences are shown in Figure S4 in File S1. Besides, 20 individual colonies of each group were sequenced as mentioned above and the indel frequency of four groups all exceeded 35% (Figure 5K and Figure S8 in File S1). There were 7, 8, 10, and 6 types of indels, respectively, documented in the sequences of the N-Cas9 protein-, C-Cas9 protein-, N-Cas9-C protein-, and N-Cas9-C mRNA-injected embryos (Figure 5L and Figure S8 in File S1). There was no significant difference in knockout efficiency between the three Cas9 protein variants and Cas9 mRNA for *gol* target 2 (all *P* > 0.05).

**Figure 6:**
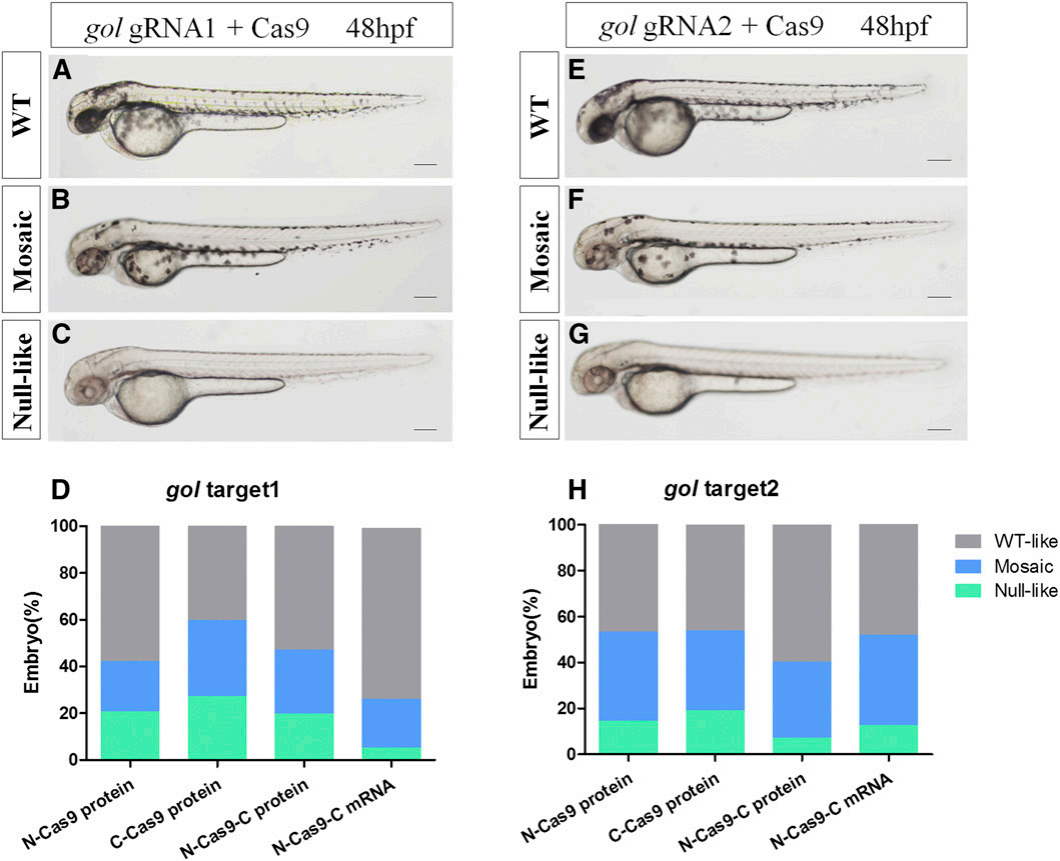
Mutagenetic phenotype of various nuclear localization signal (NLS)-fused Cas9 proteins and N-Cas9-C mRNA for two sites of the *gol* gene. (A–C) and (E–G) show lateral views of wild-type (WT) (A and E), *gol*-target 1 (B and C), and *gol*-target 2 (F and G) at 48 hpf (hr postfertilization). The phenotypes observed were WT (A and E), mosaic retinal pigmented epithelium (RPE) (B and F), and unpigmented RPE (C and G). (D and H) Proportions of embryos with each phenotype. Scale bars: 0.2 mm.

In another way, mutation of *gol* leads to hypopigmentation of the retinal pigmented epithelium and skin melanophores. Similar to *tyr*, targeting of *gol* resulted in three phenotypes: WT-like, mosaic, and null-like. The null-like phenotype had no melanin in the REF or skin melanophores (Figure 6, A–C and E–G). We analyzed the phenotypic proportion at 48 hpf for over 180 embryos; N-Cas9-C mRNA induced the lowest proportion of the null-like phenotype at *gol* target 1 (∼5%) and a higher proportion of the WT-like phenotype. N-Cas9 protein and N-Cas9-C protein both induced ∼20% of the null-like phenotype at *gol* target 1, and Cas9-NLS-C protein led to the highest proportions of the null-like and mosaic phenotypes (Figure 6D).

For *gol* target 2, N-Cas9-C protein induced the lowest proportion of the null-like phenotype, and all types of Cas9 led to similar proportions of mosaic phenotype (33.2–39.3%; Figure 6H).

### Various types of Cas9 with or without NLS-induced efficient multiplex genome mutagenesis resulted in multiple phenotypes

Since various types of NLS-fused Cas9 induced similarly high efficiency in genome editing in zebrafish, we suspected that Cas9 protein may not really require an NLS to assist import into the nucleus. To determine whether Cas9 protein without an NLS has DNA cleavage activity in zebrafish, we used another type of Cas9 protein without an NLS to target the same two sites of the *tyr* and *gol* genes under the same conditions. Interestingly, we found that Cas9 protein without an NLS induced hypopigmentation in proportions of 32.0 and 39.5% at *tyr* target 1 and *gol* target 2. However, for the *tyr* target 2 and *gol* target 1, the efficiency of T7E1 assays were much lower, consistent with a low proportions of mutation phenotypes occurring (Figure S2 in File S1).

**Figure 7:**
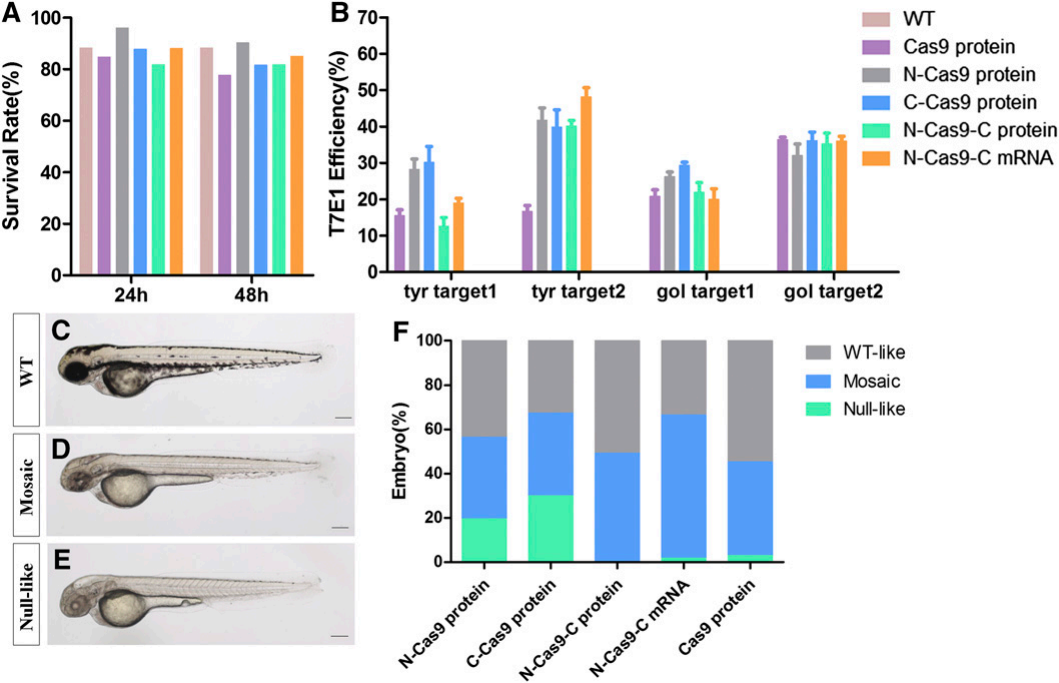
Multiplex knockout efficiency of various nuclear localization signal (NLS)-fused Cas9 proteins and N-Cas9-C mRNA. Survival rates of zebrafish embryos at 24 and 48 hr postfertilization (hpf) (A) and T7 Endonuclease I (T7E1) assays of the rates of mutagenesis for each site (B). The phenotypes observed were wild-type (WT) (C), severe mosaic hypopigmentation (D), and null-like (E). (F) Proportions of embryos with each phenotype. Scale bars: 0.2 mm.

To explore whether the five types of Cas9 had effects on multiplex genome editing, we co-injected 400 pg N-Cas9-C mRNA, 800 pg of the three NLS-fused Cas9 proteins, or 800 pg Cas9 protein without an NLS with a mixture of four gRNAs (*tyr* gRNA1, 16 pg; *tyr* gRNA2, 100 pg; *gol* gRNA1, 20 pg; and *gol* gRNA2, 100 pg) into single-cell-stage zebrafish embryos, expecting to target two genes and four target sites at the same time. The survival rates at 24 and 48 hpf were similar for each group, all >75% (Figure 7A). T7E1 assays showed that NLS-fused Cas9 was induced by 8.7–37.6% at *tyr* target 1, 31.1–53% at *tyr* target 2, 15.5–31.0% at *gol* target 1, and 26–40.7% at *gol* target 2. The efficiencies of multiplex gene editing were comparable to the efficiencies of previous targeting of single sites (Figure 7B, *tyr2* and *gol2*: *P* > 0.05, and *tyr1* and *gol1*: *P* > 0.01). However, genomic disruption rates of no NLS-fused Cas9 protein were lower at two loci of the *tyr* gene and *gol* target 1 (*tyr1*: *P* = 0.003682; *tyr2*: *P* = 0.000258; and *gol1*: *P* = 0.0319; *gol2: P* < 0.05), but were similar high at *gol* target 2, suggesting that the mutagenesis rates of no NLS-fused Cas9 proteins vary from site to site (Figure 7B). After co-injection, we observed severe pigmentation defects in the embryos, included null-like and mosaic phenotypes (Figure 7, C–F). Phenotypic analysis indicated that these five kinds of Cas9 similarly induce high hypopigmentation (Figure 7G). Collectively, both T7E1 assays and phenotypic analysis indicated that the various types of Cas9 had similar efficiencies on multiplex genome editing in zebrafish, and that the mutagenesis efficiencies of multiplex gene targeting were comparable with those of targeting a single gene.

## Discussion

A previous study of CRISPR/Cas9 technology indicated that C-NLS-Cas9 protein and *Cas9* mRNA induce similar rates of genome modifications in zebrafish founder embryos, though Cas9 protein induced a higher rate of mutagenesis than *Cas9* mRNA at the *gria3a* and *utx1* loci (Gagnon *et al.* 2014). Therefore, we sought to directly compare the targeting efficiency of Cas9 protein and *Cas9* mRNA. Our results showed that all three NLS-fused Cas9 proteins and *Cas9* mRNA resulted in high knockout efficiency for the two sites in the *tyr* and two sites in the *gol* genes. Overall, we conjecture that the knockout efficiency of gRNA/Cas9-encoding proteins or mRNA in zebrafish varies for different gRNAs.

Similarly, the survival rates of the zebrafish embryos were not significantly different for the various Cas9 proteins and *Cas9* mRNA at each of the four target sites, although the survival rates were lower for *gol* target 2 than the other three sites. Likewise, previous studies have demonstrated zinc-finger nucleases, transcription activator-like effector nucleases, and CRISPR/Cas9 targeting rates vary depending on the *loci* (Foley *et al.* 2009; Cade *et al.* 2012; Hwang *et al.* 2013). At the phenotypic level, targeting two different sites in *tyr* and *gol* resulted in similar pigmentation defects. We conclude that different gRNAs targeting the same gene will generate the same phenotype, as previously described (Jao *et al.* 2013), but that the rate of mutagenesis (at the genomic level) may not be equivalent (Li *et al.* 2014).

Targeting the *tyr* or *gol* genes using the CRISPR/Cas9 system in WT zebrafish embryos led to hypopigmentation and genomic disruption; T7E1 assays and sequencing further confirmed that all of the NLS-fused Cas9 proteins resulted in successful gene editing. In contrast, Shen *et al.* (2013) reported that Cas9 with either an N- or C-terminal-fused NLS or three N-terminal-fused NLS could not lead to functional cleavage of the genome in zebrafish embryos, and that only addition of an NLS containing a 32 amino acid linker to the N-terminus of Cas9 resulted in gene editing. Moreover, two other publications reported that *Cas9* mRNA with a C-terminal NLS and or both N- and C-terminal NLS led to gene editing in HEK293T cells (Cong *et al.* 2013; Mali *et al.* 2013). Our results indicate that the position of the NLS on Cas9 protein or mRNA does not affect the gene editing function of the CRISPR/Cas9 system in zebrafish. Hence, we hypothesized that Cas9 protein may not require an NLS to assist import into the nucleus. The Cas9 protein without an NLS induced comparable high targeted mutagenesis at *gol* target 2, partially supporting our hypothesis. In future studies, it would be interesting to examine the mechanism by which Cas9 protein without an NLS imports into the nucleus in eukaryotic organisms. Additionally, to improve the mutation rates for other gene loci, future studies should focus on the specific structures of Cas9 and gRNA that affect knockout efficiency.

T7E1 assays and the phenotypic mutation rates revealed no significant differences in the targeting rates of the three NLS-fused Cas9 proteins tested in this study. Similarly, Guilinger *et al.* (2014) fused FokI to either the N- or C-termini of an inactive variant Cas9 protein (dCas9), and varied the location of the NLS from either terminus to between the two domains; the FokI-dCas9 fusion variants led to similar frequencies. Additionally, a recent publication demonstrated that adding an NLS to the N-terminal or both the N- and C-termini of dCas9 did not improve transposition activity or gene targeting in human cells (Luo *et al.* 2017). Therefore, our results verify that the location and numbers of NLS do not affect knockout efficiency in zebrafish as the N-Cas9, C-Cas9, and N-Cas9-C proteins had similarly high knockout efficiencies.

Disruption of the endogenous genes *tyr* and *gol* led to the expected phenotypes in the F_0_ generation. When targeting *tyr* target 1, N-Cas9-C mRNA led to a higher proportion of the null-like phenotype (≤ 40%), though T7E1 assays showed that the rate of mutagenesis was highest for N-Cas9-C protein and *tyr* gRNA1 (Figure 3D). When targeting *gol* target 1, C-Cas9 protein induced the highest proportions of the null-like and mosaic phenotypes, though T7E1 assays showed that the mutagenesis rate was highest for N-Cas9-C protein and gRNA2. Similarly, we suggest that a high phenotypic mutation rate may not always reflect a high mutagenesis rate in the T7E1 assay. We speculate that this phenomenon occurs for two reasons. First, as these endogenous genes related to body pigmentation are dominant, cleavage of both alleles will result in an obvious hypopigmentation phenotype. Second, T7E1 assays cannot detect mutations if single mutated DNA alleles anneal together (Kim *et al.* 2014). Therefore, the mutation rate determined by the T7E1 assay may be lower than the actual mutagenesis rate.

Overall, sequencing results showed high frequencies of indels observed in the target sites (*tyr1* > 66%, *tyr2* > 73%, *gol1* > 50%, and *gol2* > 35%). Both T7E1 assays and phenotypic analysis indicated that the various NLS-fused Cas9 proteins and Cas9 mRNA had efficient effects on multiplex genome editing in zebrafish, and that the mutagenesis efficiencies of multiplex genes targeting were comparable with that of a single gene. In summary, these results indicate that gRNA/Cas9-encoding proteins lead to a high indel frequency, regardless of the position of the NLS fusion. Furthermore, Cas9 protein and *Cas9* mRNA result in similarly high knockout efficiencies with single gRNA or multiple gRNAs. These data may help researchers to select various NLS-fused Cas9 when using the CRISPR/Cas9 gene editing system.

## Supplementary Material

Supplemental material is available online at www.g3journal.org/lookup/suppl/doi:10.1534/g3.117.300359/-/DC1.
