# Supplementary material for: Comparison of Various Nuclear Localization Signal-Fused Cas9 Proteins and Cas9 mRNA for Genome Editing in Zebrafish
Source: G3 (Bethesda). 2018 Jan 2;8(3):823–31. doi: 10.1534/g3.117.300359 (PMC5844304; doi:10.1534/g3.117.300359)
Supplement: Supplementary file 1 [file 823FileSI.pdf]

Supplementary Information

Comparison of various nuclear localization signal-fused Cas9 proteins and *Cas9* mRNA for genome editing in zebrafish

Peinan Hu[1、2、3], Xueying Zhao[1、2、3],Qinghua Zhang[1、2、3], Weiming Li[1、4], Yao Zu[1、2、3].

1. International Research Center for Marine Biosciences, Ministry of Science and Technology, College of Fisheries and Life Sciences, Shanghai Ocean University, Shanghai, 201306, China;
2. Key Laboratory of Exploration and Utilization of Aquatic Genetic Resources, Ministry of Education, College of Fisheries and Life Sciences, Shanghai Ocean University, Shanghai, 201306, China;
3. Key Laboratory of Freshwater Aquatic Genetic Resources, Ministry of Agriculture, College of Fisheries and Life Sciences, Shanghai Ocean University, Shanghai, 201306, China
4. Department of Fisheries and Wildlife, Michigan State University, East Lansing, MI 48824, USA

Corresponding author: Yao Zu (Email: yzu@shou.edu.cn)

Figure S1

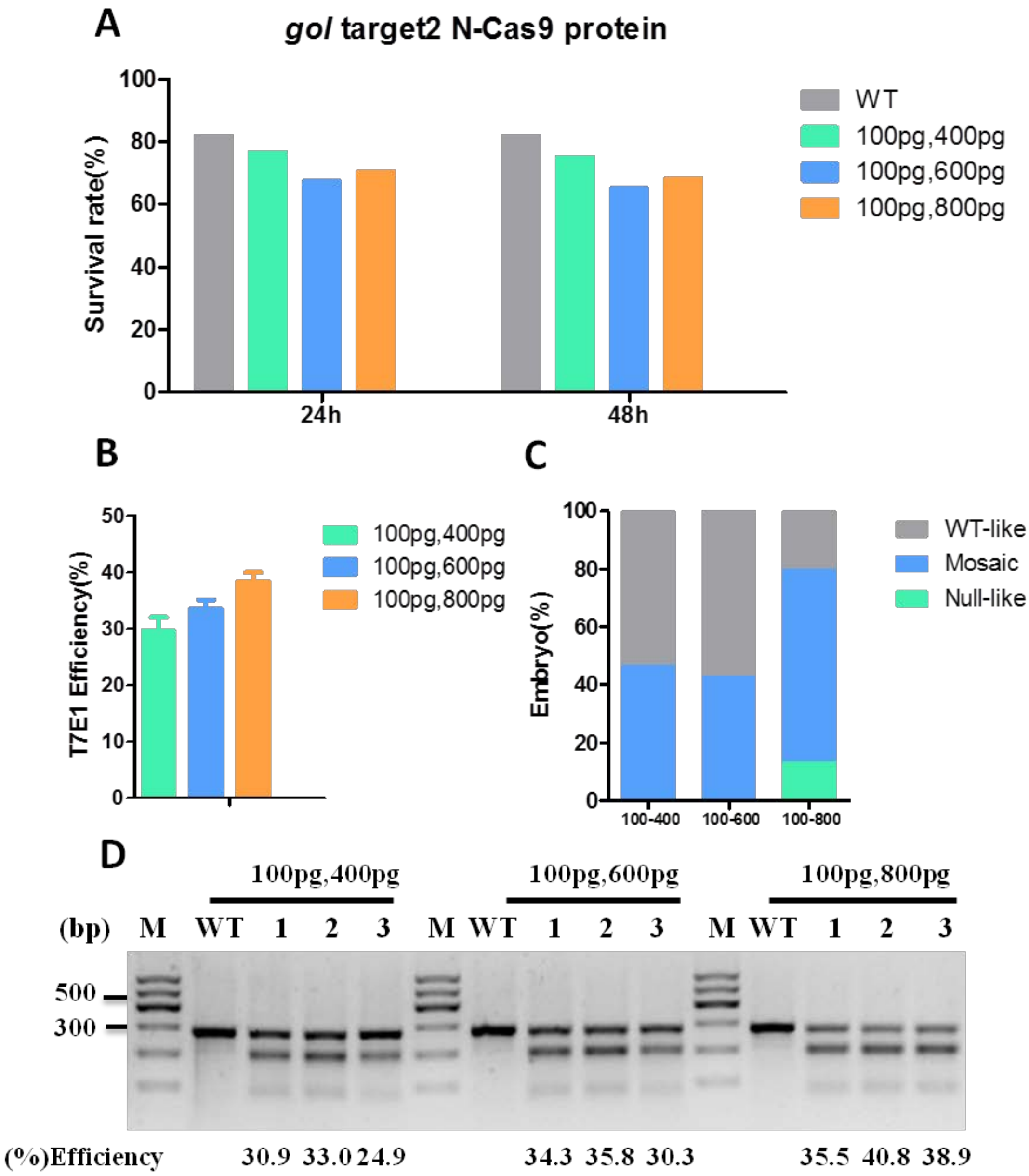

Figure S1 Survival rates of zebrafish embryos injected N-NLS-fused Cas9 protein and 100pg of gRNA targeting the *gol* target2 (A), and T7E1 assays of the rates of mutagenesis (B and D, $P < 0.05$) and proportions of embryos with each phenotype (C) for *gol* target 2.

Figure S2

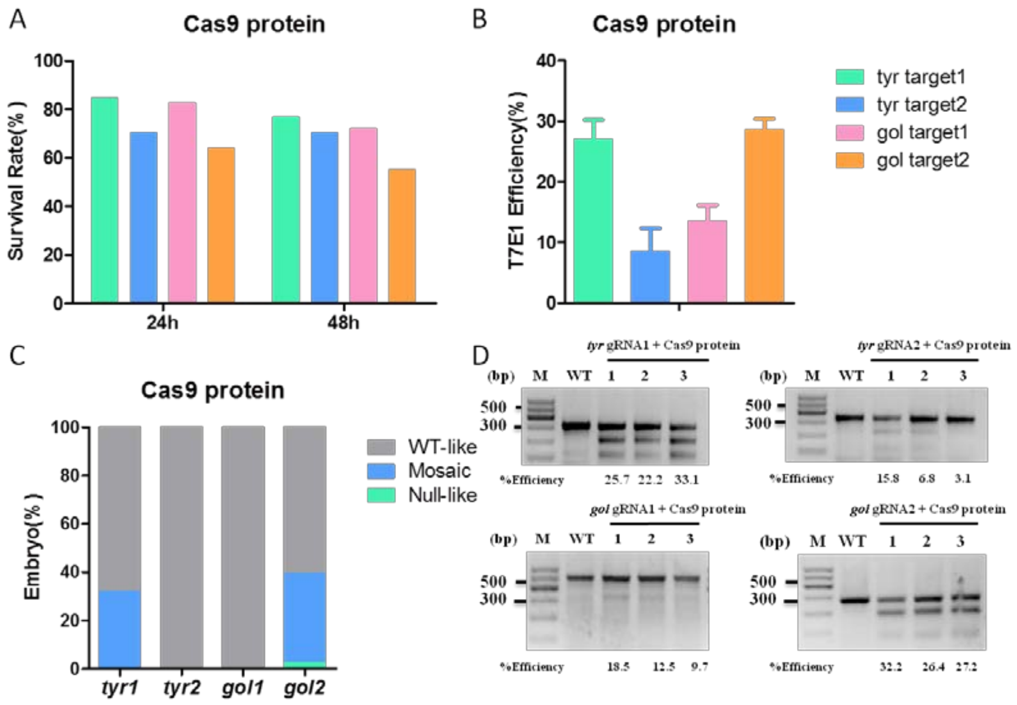

Figure S2 Knockout efficiency of the Cas9 proteins without NLS for four loci of *tyr* and *gol* gene. Survival rates of zebrafish embryos(A) and T7E1 assays of the rates of mutagenesis for each loci(B and D). (C) Proportions of embryos with each phenotype.

Figure S3

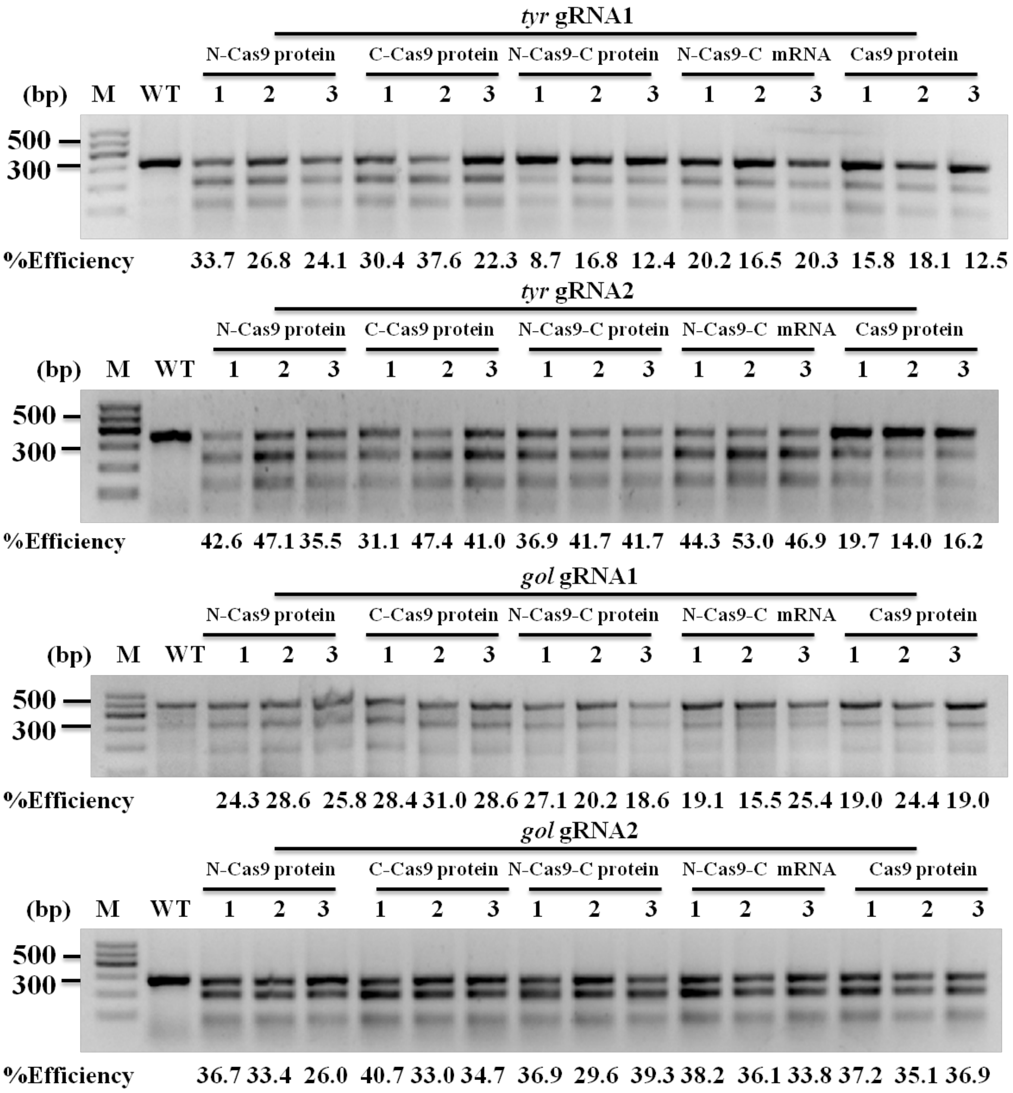

Figure S3 Multiplex Genome Mutation efficiency of the various NLS-fused Cas9 proteins and Cas9 mRNA for four loci of *tyr* and *gol* gene. T7E1 assays showed the rates of mutagenesis for *tyr* target 1 (A) , *tyr* target2 (B), *gol* target1 (C) and *gol* target 2 (D).

Figure S4

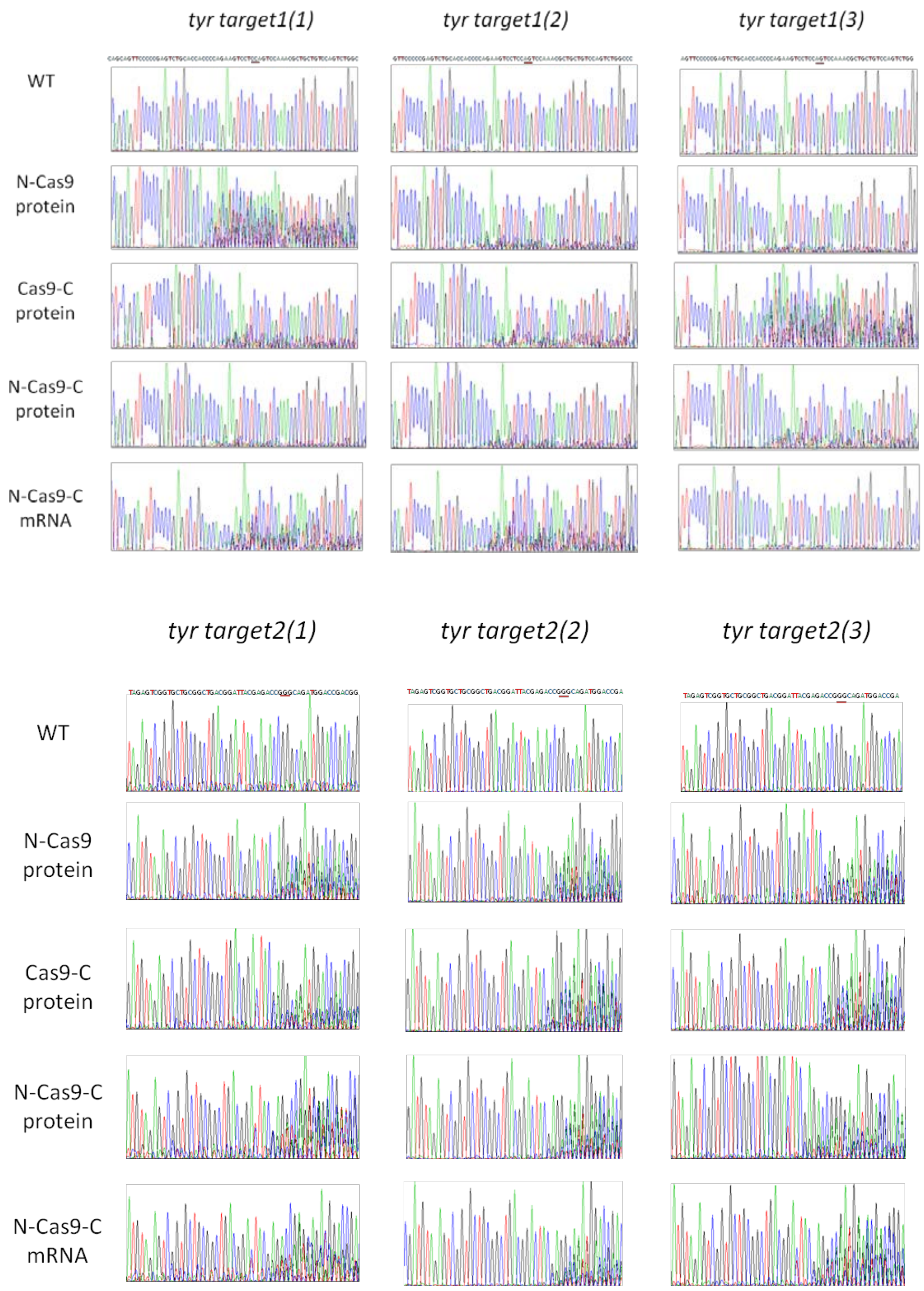

tyr target1(1)
tyr target1(2)
tyr target1(3)
WT
N-Cas9 protein
Cas9-C protein
N-Cas9-C protein
N-Cas9-C mRNA
tyr target2(1)
tyr target2(2)
tyr target2(3)
WT
N-Cas9 protein
Cas9-C protein
N-Cas9-C protein
N-Cas9-C mRNA

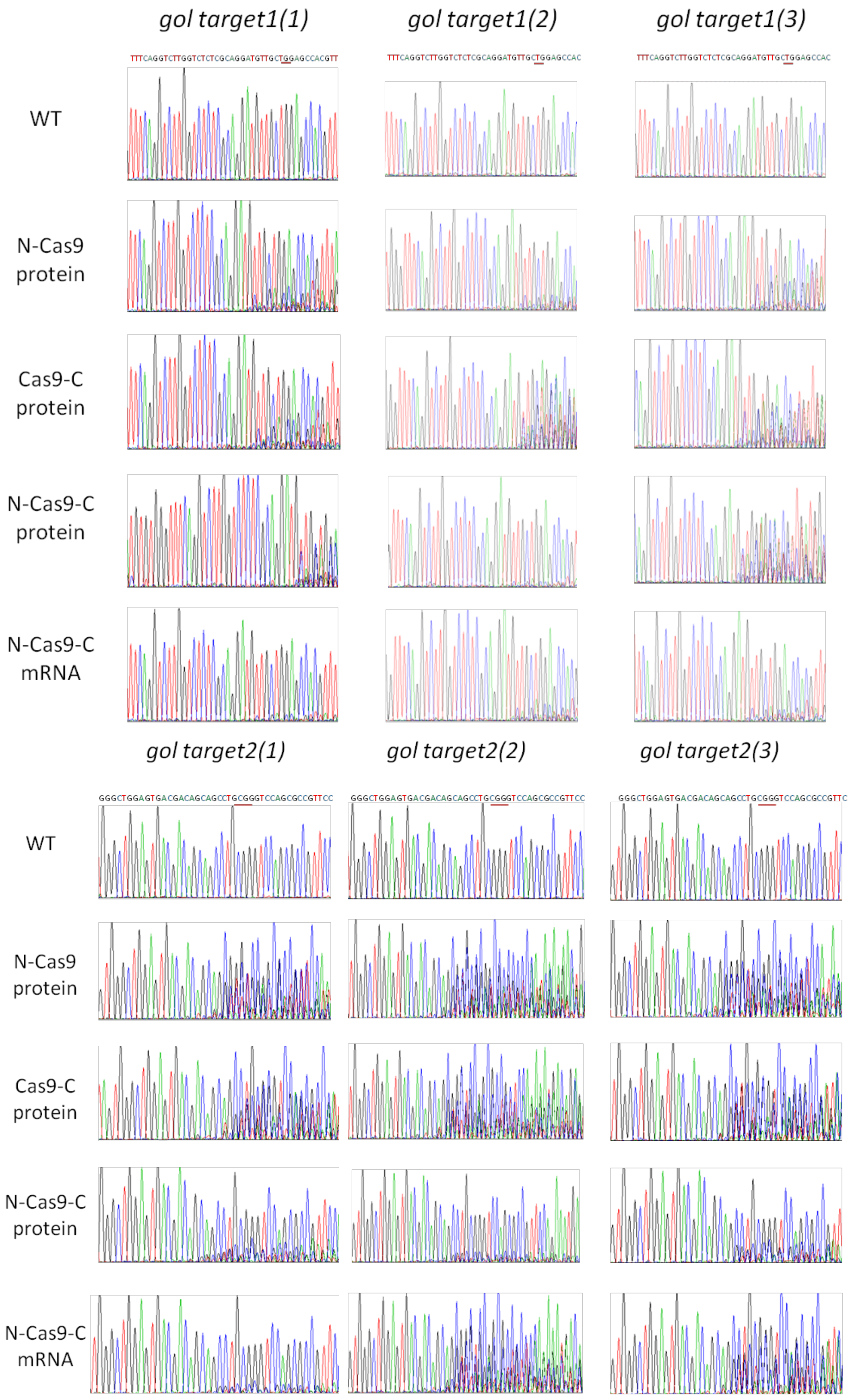

Figure S4 The PCR Sanger sequencing at two *loci* of *tyr* and two *loci* of *gol*.

Figure S5

***tyr* target1:**

N-Cas9 protein：(indel:12,WT:5)

WT： TCGTCTCTCCAGCAGTTCCCCGAGTCTGCACCACCCCAGAAGTCCTCCAGTCCAA

N1： TCGTCTCTCCAGCAGTTCCC--TCGTCTCT------CCAGAAGTCCTCCAGCCCAA(-11bp,+2bp)

N2： TCGTCTCTCCAGCAGT----------------------------CCTCCAGTCCAA(-28bp)

N3： TCGTCTCTCCAGCAGTTCCCCGAGTCTGCACC-----A-----CCTCCAGTCCAA(-10bp)

N4： TCGTCTCTCCAGCAGTTCCCCGAGTCTGCACCACCCACAGAAGTCCTCCAGTCCAA(+1bp)

N5： GCAGTTCCCCGAGTCTGCACCACCGAGTCTGCACCACCACCCAGAAGTCCTCCAGT(+16bp)

N6： TCGTCTCTCCAGCAGCTCCCCGAGTCTGCACCTCC-------------AGTCCAA(-13bp)

N7： TCGTCTCTCCAGCAGTTCCCCGAGTCTGCACCACCCCAGAAGTCCTCCAGTCCAA(WT)

N8： TCGTCTCTCCAGCAGTTCCCCGAGTCGGCACCACCCAAGTCCAAGTCCTCCAGTCC(-2bp,+5bp)

N9: TCGTCTCTCCAGCAGTTCCCCGAGTCTGCACCACCCCAGAAGTCCTCCAGTCCAA(WT)

N10: TCGTCTCTCCAGCAGTTCCCCGAGTCTGCACCACCCCAGAAGTCCTCCAGTCCAA(WT)

N11: TCGTCTCTCCAGCAGT----------------------------CCTCCAGTCCAA(-28bp)

N12: TCGTCTCTCCAGCAGTTCCCCTGAGTCTGCA------CAGAAGTCCTCCAGTCCAA(-6bp)

N13: GTCTCTCCAGCAGTTCCCCGAGTCTGCACCACCCTCCAGAAGTCCTCCAGTCCAA(+2bp)

N14: TTCCCCCGAGTCTGCACCACCCGAAGTCCTCCACCACCAGAAGTCCTCCAGTCCAA(-1bp,+17bp)

N15: TCCAGCAGTTCCCCGAGTCTGCACCACCCTCCAGTCCAGAAGTCCTCCAGTCCAA(+7bp)

N16: TCGTCTCTCCAGCAGTTCCCCGAGTCTGCACCACCCCAGAAGTCCTCCAGTCCAA(WT)

N17: TCGTCTCTCCAGCAGTTCCCCGAGTCTGCACCACCCCAGAAGTCCTCCAGTCCAA(WT)

C-Cas9 protein：(indel:14,WT:4)

WT： TCGTCTCTCCAGCAGTTCCCCGAGTCTGCACCACCCCAGAAGTCCTCCAGTCCAA

C1： TCGTCTCTCCAGCAGTTCCCCGAGTCTGCACCACC------------------AA(-18bp)

C2： TCGTCTCTCCAGCAGTTCCCCGAGTCTGCACCACCCCAGAAGTCCTCCAGTCCAA(WT)

C3： TCGTCTCTCCAGCAGTTCCCCGAGTCTGCACCACCCCAGAAGTCCTCCAGTCCAA(WT)

C4： TCGTCTCTCCAGCAGTTCCCCGAGTCTGCACCACCCCAGAAGTCCTCCAGTCCAA(WT)

C5： TCGTCTCTCCAGCAGTTCCCCGAGTCTGCA--------GAAGTCCTCCAGTCCAA(-8bp)

C6： TCGTCTCTCCAGCAGTTCCCCGAGTCTGCACCACC----------TCCAGTCCAA(-10bp)

C7： TCGTCTCTCCAGCAGTTCCCCGAGTCTGCACCACCCTCC—AGTCCTCCAGTCCAA(-3bp,+2bp)

C8： C------AG---CAGAGTGTAAAGCCTCTCCGGTGTGTGTGAGTCCTCCAGTCCAA(-32bp,+31bp)

C9： CTCTCCAGCAGTTCCCCGAGTCTGCACCACCCTCC----AAGTCCTCCAGTCCAA(-4bp,+4bp)

C10: TCGTCTCTCCAGCAGTTCCCCGAGTCTGCACCACCC---------TCCAGTCCAA(-9bp)

C11: TCGTCTCTCCAGCAGTTCCCCGAGTCTGCACC-------------TCCAGTCCAA(-13bp)

C12: TCGTCTCTCCAGCAGTTCCCCGAGTCTGCACC-------------TCCAGTCCAA(-13bp)

C13: TCGTCTCTCCAGCAGTTCCCCGAGTCTGCA--------GAAGTCCTCCAGTCCAA(-8bp)

C14: TCGTCTCTCCAGCAGTTCCCCGAGTCTGCACCA-----GAAGTCCTCCAGTCCAA(-5bp)

C15: TCGTCTCCAGCAGTTCCCCGAGACCTGCA-----CCAGAAGTCCTCCAGTCCAA(-6bp,+2bp)

C16: TCGTCTCTCCAGCAGTTCCCCGAGTCTGCACCA----------------GTCCAA(-16bp)

C17: CTCTCCAGCAGTTCCCCGAGTCTGCACCACCCTCC----AAGTCCTCCAGTCCAA(-4bp,+4bp)

C18: TCGTCTCTCCAGCAGTTCCCCGAGTCTGCACCACCCCAGAAGTCCTCCAGTCCAA(WT)

N-Cas9-C protein：(indel:16,WT:3)

WT： TCGTCTCTCCAGCAGTTCCCCGAGTCTGCACCACCCCAGAAGTCCTCCAGTCCAA

B1： TCGTCTCTCCAGCAGTTCCCCGAGTCTGCACCACCT-AGAAGTCCTCCAGTCCAA(-2bp,+1bp)

B2： TCGTCTCTCCAGCAGTTCCCCGAGTCTGCA--------GAAGTCCTCCAGTCCAA(-8bp)

B3： TCGTCTCTCCAGCAGTTCCCCGAGTCTGCA--------GAAGTCCTCCAGTCCAA(-8bp)

B4： TCGTCTCTCCAGCAGTTCCCCGAGTCTGCACCACC-----AGTCCTCCAGTCCAA(-5bp)

B5： TCGTCTCTCCAGCAGTTCCCCGAGTCTGCACCACC--AGAAGTCCTCCAGTCCAA(-2bp)

B6： TCGTCTCTCCAGCAGTTCCCCGAGTCTGCACCA-------AGTCCTCCAGTCCAA(-7bp)

B7： CAGCAGTTTCCCCGAGTCTGCACCACCACCACCAGAAGTCCTCCAGTCCAGTCCAA(+4bp)

B8： AGTTCCCCCGAGTCTGCACCA-----GAAGTCCTCCAGTCCAAACGCTGCTGTCCAA(-5bp)

B9： CGTCTCTCCAGCAGTTCCCCGAGTCTGCACCAC---AGAAGTCCTCCAGTCCAA(-3bp)

B10: TCGTCTCTCCAGCAGTTCCCCGAGTCTGCACCA-----GAAGTCCTCCAGTCCAA(-5bp)

B11: TCGTCTCTCCAGCAGTTCCCCGAGTCTGCA--------GAAGTCCTCCAGTCCAA(-8bp)

B12: TCGTCTCTCCAGCAGTTCCCCGAGTCTGCACCACCCCAGAAGTCCTCCAGTCCAA(WT)

B13: GTCTCTCTCCAGCAGTTCCCCGAGTCTGCACCA-----GAAGTCCTCCAGTCCAA(-5bp)

B14: TCGTCTCTCCAGCAGTTCCCCGAGTCTGCACCACCCCAGAAGTCCTCCAGTCCAA(WT)

B15: TCGTCTCTCCAGCAGTTCCCCGAGTCTGCACCAGA--AGAAGTCCTCCAGTCCAA(-4bp,+2bp)

B16: TCGTCTCTCCAGCAGTTCCCCGAGTCTGCACCA-----GAAGTCCTCCAGTCCAA(-5bp)

B17: TCGTCTCTCCAGCAGTTCCCCGAGTCTGCACCACCC---------TCCAGTCCAA(-9bp)

B18: TCGTCTCTCCAGCAGTTCCCCGAGTCTGCACCGAGTCCAGAAGTCCTCCAGTCCAA(-2bp,+3bp)

B19: TCGTCTCTCCAGCAGTTCCCCGAGTCTGCACCACCCCAGAAGTCCTCCAGTCCAA(WT)

N-Cas9-C mRNA：(indel:12,WT:6)

WT： TCGTCTCTCCAGCAGTTCCCCGAGTCTGCACCACCCCAGAAGTCCTCCAGTCCAA

m1： TCGTCTCTCCAGCAGTTCCCCGAGTCTGCACCACCCCAGAAGTCCTCCAATCCAA(-1bp,+1bp)

m2： TCGTCTCTCCAGCAATTCCCCGAGTCTGCA-------------------GTCCAA(-19bp)

m3： TCGTCTCTCCAGCAGTTCCCCGAGTCTGCACCT-CCCAGAAGTCCTCCAGTCCAA(-3bp,+2bp)

m4： TCGTCTCTCCAGCAGTTCCCCCGAGTCTGCACCTCCCCAGAAGTCCTCCAGTCCAA(-1bp,+2bp)

m5： TCGTCTCTCCAGCAGTTCCCCGAGTCTGCACCACCCCAGAAGTCCTCCAGTCCAA(WT)

m6： GCAGTTCCCCGAGTCTGCACC----------TCC----------------CTGTC(-26bp)

m7： TCGTCTCTCCAGCAGTTCCCCGAGTCTGCACCACCCCAGAAGTCCTCCAGTCCAA(WT)

m8： TCCCCCGAGTCTGCACCACCCTGCACCACAAGAGTCTGCACCACAAGTCCTCCAGT(-21bp,+1bp)

m9： TCGTCTCTCCAGCAGTTCCCCGAGTCTGCACCACCCCAGAAGTCCTCCAGTCCAA(WT)

m10: TCGTCTCTCCAGCAGTTCCCCGAGTCTGCACCACCCCAGAAGTCCTCCAGTCCAA(WT)

m11: TCGTCTCTCCAGCAGTTCCCCGAGTCTGCACCACC--AGAAGTCCTCCAGTCCAA(-2bp)

m12: GTCTCTCCAGCAGTTCCCCGAGTCTGCACCACCCGGCAGAAGTCCTCCAGTCCAA(+2bp)

m13: TCGTCTCTCCAGCAGTTCCCCGAGTCTGCACCACCCCAGAAGTCCTCCAGTCCAA(WT)

m14: GTCTCTCCAGCGGTTCCCCGAGTCTGCACCACCCTCCAGAAGTCCTCCAGTCCAA(+2bp)

m15: TCGTCTCTCCAGCAGTTCCCCGAGTCTGCACCAG----GAAGTCCTCCAGTCCAA(-4bp)

m16: TCTCCAGCAGTTCCCCGAGTCTGCACCACCCCAGAAGTCCTCCAGTCCAA(WT)

m17: TCGTCTCTCCAGCAGTTCCCCGAGTCTGCACCACCTCC--AGTCCTTCAGTCCAA(-3bp,+1bp)

m18: TCGTCTCTCCAGCAGTTCCCCGAGTCTGCACCACC-------------AGTCCAA(-13bp)

Figure S6

***tyr* target2:**

N-Cas9 protein：(indel:19,WT:0)

WT：AGTCGGTGCTGCGGCTGACGGACTACGAGACCGGGCAGATGGACCGACGGGCAAACCTG

N1：AGTCGGTGCTGCGGCTGACGGACTACGAGCA--GGCAGATGGACCGACGGGCAAACCTG(-4bp,+2bp)

N2：AGTCGGTGCTGCGGCTGACGGACTAC-----CGGGCAGATGGACCGACGGGCAAACCTG(-5bp)

N3：TCGGTGCTGCGGCTGACGGACTACGACTACCGGGCAGATGGACCGACGGGCAAACCTG(-1bp,+2bp)

N4：--CAGATGGAC(-65bp)

N5：AGTCGGTGCTGCGGCTGACGGACTAC-----CGGGCAGATGGACCGACGGGCAAACCTG(-5bp)

N6：AGTCGGTGCTGCGGCTGACGGACTACG------GGCAAATGGACCGACGGGCAAACCTG(-6bp)

N7：AGTCGGTGCTGCGGCTGACGGACTACGA----GGGCAGATGGACCGACGGGCAAACCTG(-4bp)

N8：AGTCGGTGCTGCGGCTGACGGACTAC-----CGGGCAGATGGACCGACGGGCAAACCTG(-5bp)

N9：AGTCGGTGCTGCGGCTGACGGACTACGA------GCAGATGGACCGACGGGCAAACCTG(-6bp)

N10:AGTCGGTGCTGCGGCTGACGGACTACGA------GCAGATGGACCGACGGGCAAACCTG(-6bp)

N11:AGTCGGTGCTGCGGCTGACGGACTACGA------GCAGATGGACCGACGGGCAAACCTG(-6bp)

N12:AGTCGGTGCTGCGGCTGACGGA---------------------CCGACGGGCAAACCTG(-21bp)

N13:AGTCGGTGCTGCGGCTGACGGACTACGA------GCAGATGGACCGACGGGCAAACCTG(-6bp)

N14:AGTCGGTGCTGCGGCTGACGGA---CT------------------ACGGGCAAACCTG(-22bp)

N15:GGTGCTGCGGCTGACGGACTACGAGGACTACCGGGCAGATGGACCGACGGGCAAACCTG(+4bp)

N16:AGTCGGTGCTGCGGCTGACGGACTAC-----CGGGCAGATGGACCGACGGGCAAACCTG(-5bp)

N17:GGTGCTGCGGCTGACGGACTACGACGACTACCGGGCAGATGGACCGACGGGCAAACCTG(-1bp,+5bp)

N18:GTGCTGCGGCTGACGGACTACGA-----GGCAGATGGACCGACGGGCAAACCTGAGCTG(-5bp)

N19:GTGCTGCGGCTGACGGACTACGA-----GGCAGATGGACCGACGGGCAAACCTGAGCTG(-5bp)

C-Cas9 protein: (indel:18,WT:0)

WT：GAGTCGGTGCTGCGGCTGACGGACTACGAGACCGGGCAGATGGACCGACGGGCAAACCTG

C1：GTGCTGCGGCTGACGGATTACGAGCAGATGGACGGGCAGATGGACCGACGGGCAAACCTG(-1bp,+7bp)

C2：GAGTCGGTGCTGCGGCTGACGGATTACGAG-----GCAGATGGACCGACGGGCAAACCTG(-5bp)

C3：GAGTCGGTGCTGCGGCTGACGGACTAC--GACCGGGCAGATGGACCGACGGGCAAACCTG(-2bp)

C4：GAGTCGGTGCTGCGGCTGACGGATTAC-----CGGGCAGATGGGCCGACGGGCAAACCTG(-5bp)

C5：GAGTCGGTGCTGCGGCTGACGGA---CT--ACCGGGCAGATGGACCGACGGGCAAACCTG(-6bp,+1bp)

C6：GAGTCGGTGCTGC-------------------CGGGCAGATGGACCGACGGGCAAACCTG(-19bp)

C7：ATTACGATCTCTCGCATATTTGATAGCTTAACCGGGCAGATGGACCGACGGGCAAACCTG(+22bp)

C8：CTGC--GGATCCGGGT-----------------------------------ACCGGGCAG(-40bp,+2bp)

C9： AGTCGGTGCTGCGGCTGACGG-------------GCAGATGGACCGACGGGCAAACCTG(-13bp)

C10:GTCGGTGCTGCGGCTGACGGACTACGAGTAGCTA----------CGACGGGCAAACCTG(-10bp,+5bp)

C11:AGTCGGTGCTGCGGCTGACGGACTA------CGGGCAGATGGACCGACGGGCAAACCTG(-6bp)

C12:GAGTCGGTGCTGCGGCTGACGGA---CT--ACCGGGCAGATGGACCGACGGGCAAACCTG(-5bp)

C13:AGTCGGTGCTGCGGCTGACGGACTACG-GACCGGGCAGATGGACCGACGGGCAAACCTG(-1bp)

C14:AGTCGGTGCTGCGGCTGACGGACTACGA-----GGCAGATGGACCGACGGGCAAACCTG(-5bp)

C15:AGTCGGTGCTGCGGCTGACGGACTACGA------GCAGATGGACCGACGGGCAAACCTG(-6bp)

C16:TGCTGCGGCTGACGGACTACGATGGGCAGACCGGGCAGATGGACCGACGGGCAAACCTG(+6bp)

C17:GAGTCGGTGCTGCGGCTGACGGACTACGAGAT--GGCAGATGGACCGACGGGCAAACCTG(-3bp,+1bp)

C18:GAGTCGGTGCTGCGGCTGACGGACTACT--ACCGGGCAGATGGACCGACGGGCAAACCTG(-3bp,+1bp)

N-Cas9-C protein：(indel:14,WT:5)

WT：GAGTCGGTGCTGCGGCTGACGGACTACGAGACCGGGCAGATGGACCGACGGGCAAACCTG

B1： TGACGGACTACGAGGTACCGCGGCTGCCCACCGGGCAGATGGACCGACGGGCAAACCTG(+15bp)

B2： GAGTCGGTGCTGCGGCTGACGGA---CT—ACCGGGCAGATGGACCGACGGGCAAACCTG(-5bp)

B3： GCTGCGGCTGACGGACTACGAGCAGATGGACCGGGCAGATGGACCGACGGGCAAACCTG(+7bp)

B4： AGTCGGTGCTGCGGCTGACGGACTACG---------AGATGGACCGACGGGCAAACCTG(-9bp)

B5： AGTCGGTGCTGCGGCTGACGGACTACG-----GGGCAGATGGACCGACGGGCAAACCTG(-5bp)

B6： AGTCGGTGCTGCGGCTGACGGACTACGAGACCGGGCAGATGGACCGACGGGCAAACCTG(WT)

B7： AGTCGGTGCTGCGGCTGACGGATTACGAG-----GCAGATGGACCGACGGGCAAACCTG(-5bp)

B8： GAGTCGGTGCTGCGGCTGACGGA---CT--ACCGGGCAGATGGACCGACGGGCAAACCTG(-5bp)

B9： AGTCGGTGCTGCGGCTGAC------------CGGGCAGATGGACCGACGGGCAAACCTG(-12bp)

B10:GAGTCGGTGCTGCGGCTGACGGACTACGAGACCGGGCAGATGGACCGACGGGCAAACCTG(WT)

B11:GGTGCTGCGGCTGACGGACTACGCCGGGCAGATGGGCAGATGGACCGACGGGCAAACCTG(-5bp,+10bp)

B12:GAGTCGGTGCTGCGGCTGACGGACTACGA----GGGCAGATGGACCGACGGGCAAACCTG(-4bp)

B13:GAGTCGGTGCTGCGGCTGACGGACTACGAGACCGGGCAGATGGACCGACGGGCAAACCTG(WT)

B14:GAGTCGGTGCTGCGGCTGACGGACTACGAGACCGGGCAGATGGACCGACGGGCAAACCTG(WT)

B15:GAGTCGGTGCTGCGGCTGACGGA---CT--ACCGGGCAGATGGACCGACGGGCAAACCTG(-5bp)

B16:GAGTCGGTGCTGCGGCTGACGGACTACGAGACCGGGCAGATGGACCGACGGGCAAACCTG(WT)

B17:GAGTCGGTGCTGCGGCTGACGGA---CT--ACCGGGCAGATGGACCGACGGGCAAACCTG(-5bp)

B18:GAGTCGGTGCTGCGGCTGACGGA---CC--ACCGGGCAGATGGACCGACGGGCAAACCTG(-6bp,+1bp)

B19:GAGTCGGTGCTGCGGCTGACGGACTACGAGTCCGGGCAGATGGACCGACGGGCAAACCTG(-1bp,+1bp)

N-Cas9-C mRNA：(indel:14,WT:5)

WT：GAGTCGGTGCTGCGGCTGACGGACTACGAGACCGGGCAGATGGACCGACGGGCAAACCTG

m1：GAGTCGGTGCTGCGGCTGACGGACTACGAGACCGGGCAGATGGACCGACGGGCAAACCTG(WT)

m2：GAGTCGGTGCTGCGGCTGACGGACTACGAGT------AGATGGACCGACGGGCAAACCTG(-6bp)

m3：GAGTCGGTGCTGCGGCTGACGGACTACGA------GCAGATGGACCGACGGGCAAACCTG(-6bp)

m4：GAGTCGGTGCTGCGGCTGACGGACTACGAGACCGGGCAGATGGACCGACGGGCAAACCTG(WT)

m5：GAGTCGGTGCTGCGGCTGACGGACTACGA-----GGCAGATGGACCGACGGGCAAACCTG(-5bp)

m6：GAGTCGGTGCTGCGGCTGACGGACTACGA-----GGCAGATGGACCGACGGGCAAACCTG(-5bp)

m7：GAGTCGGTGCTGCGGCTGGCGGACTACGA------GCAGATGGACCGACGGGCAAACCTG(-7bp,+1bp)

m8：GAGTCGGTGCTGCGGCTGACGGACTACGACTACCGGGCAGATGGACCGACGGGCAAACCTG(-1bp,+2bp)

m9:GAGTCGGTGCTGCGGCTGACGGACTACGAGACCGGGCAGATGGACCGACGGGCAAACCTG(WT)

m10:GCGGCTGACGGACTACGATGGACTACTAAACACCGGGCAGATGGACCGACGGGCAAACCT(+13bp)

m11:GAGTCGGTGCTGCGGCTGACGGACTACGAGACCGGGCAGATGGACCGACGGGCAAACCTG(WT)

m12:GAGTCGGTGCTGCGGCTGACGGACTACGAGACCGGGCAGATGGACCGACGGGCAAACCTG(WT)

m13:GAGTCGGTGCTGCGGCTGACGGATTACGA------GCAGATGGACCGACGGGCAAACCTG(-6bp)

m14:ACGGACTACGAGATGGACCGACGGTCCATCACCGGGCAGATGGACCGACGGGCAAACCTG(+18bp)

m15:GAGTCGGTGCTGCGGCTGACGGACTAC-----------GATGGACCGACGGGCAAACCTG(-11bp)

m16:GAGTCGGTGCTGCGGCTGACGG-------------GCAGATGGACCGACGGGCAAACCTG(-13bp)

m17:CGGACTACGAGTTATTGAAAATCTGAGGTAAATCAAATCGGCAGATGGACCGACGGGCAA(-4bp,+28bp)

m18:GAGTCGGTGCTGCGGCTGACGGACTACGT-------CAGATGGACCGACGGGCAAACCTG(-8bp,+1bp)

m19:ACAGCGGCTGACGTAGAACGAGGCAGATGGACCGGGCAGATGGACCGACGGGCAAACCTG(-2bp,+10bp)

Figure S7

***gol* target1:**

N-Cas9 protein：(indel:12,WT:5)

WT：TTCAGGTCTTGGTCTCTCGCAGGATGTTGCTGGAGCCACGTTTATGGCTGCGGGGAGTTCG

N1：TTCAGGTCTTGGTCTCTCGCAGGATG---CTGGAGCCACGTTTATGGCTGCGGGGAGTTCG(-3bp)

N2：TTCAGGTCTTGGTCTCTCGCAGGATGTTGCTGGAGCCACGTTTATGGCTGCGGGGAGTTCG(WT)

N3：TTCAGGTCTTGGTCTCTCGCAGGATG---CTGGAGCCACGTTTATGGCTGCGGGGAGTTCG(-3bp)

N4：TTCAGGTCTTGGTCTCTCGC----------TGGAGCCACGTTTATGGCTGCGGGGAGTTCG(-10bp)

N5：TCTTGGTCTCTCGCAGG-TCTCTCTCTTGCTGGAGCCACGTTTATGGCTGCGGGGAGTTCG(-2bp,+7bp)

N6：TTCAGGTCTTGGTCTCTCGCAGGATGTTGCTGGAGCCACGTTTATGGCTGCGGGGAGTTCG(WT)

N7:TTCAGGTCTTGGTCTCTCGCAGGATGTTGCTGGAGCCACGTTTATGGCTGCGGGGAGTTCG(WT)

N8:TTCAGGTCTTGGTCTCTCGCAGGATG------GAGCCACGTTTATGGCTGCGGGGAGTTCG(-6bp)

N9:TTCAGGTCTTGGTCTCTCGCAGGATGTTGCTGGAGCCACGTTTATGGCTGCGGGGAGTTCG(WT)

N10:TTCAGGTCTTGGTCTCTCGCAGGATG---CTGGAGCCACGTTTATGGCTGCGGGGAGTTCG(-3bp)

N11:TTCAGGTCTTGGTCTCTCGCAGGATGTTG---GAGCCACGTTTATGGCTGCGGGGAGTTCG(-3bp)

N12:TTCAGGTCTTGGTCTCTCGCAGGATG------GAGCCACGTTTATGGCTGCGGGGAGTTCG(-6bp)

N13:TTCAGGTCTTGGTCTCTCGCAG-------------CCACGTTTATGGCTGCGGGGAGTTCG(-13bp)

N14:TTCAGGTCTTGGTCTCTCGCAGGATG---CTGGAGCCACGTTTATGGCTGCGGGGAGTTCG(-3bp)

N15:TTCAGGTCTTGGTCTCTCG-------------GAGCCACGTTTATGGCTGCGGGGAGTTCG(-13bp)

N16:TTCAGGTCTTGGTCTCTCGCAGGATGTTGCTGGAGCCACGTTTATGGCTGCGGGGAGTTCG(WT)

N17:TTCAGGTCTTGGTCTCTCGCAGGATG---CTGGAGCCACGTTTATGGCTGCGGGGAGTTCG(-3bp)

C-Cas9 protein：(indel:16,WT:1)

WT：TTCAGGTCTTGGTCTCTCGCAGGATGTTGCTGGAGCCACGTTTATGGCTGCGGGGAGTTCG

C1：CTCTCGCAGGATGTGTATTATGCAGGATGCTGGAGCCACGTTTATGGCTGCGGGGAGTTCG(+13bp)

C2：CTCTCGCAGGATGTGTATTATGCAGGATGCTGGAGCCACGTTTATGGCTGCGGGGAGTTCG(+13bp)

C3：TTCAGGTCTTGGTCTCTCGCAGGATG---CTGGAGCCACGTTTATGGCTGCGGGGAGTTCG(-3bp)

C4：TTCAGGTCTTGGTCTCTCGCAGGATG---CTGGAGCCACGTTTATGGCTGCGGGGAGTTCG(-3bp)

C5：TTCAGGTCTTGGTCTCTCGCAGGATG---CTGGAGCCACGTTTATGGCTGCGGGGAGTTCG(-3bp)

C6：TTCAGGTCTTGGTCTCTCGCAGGATGT-GCTGGAGCCACGTTTATGGCTGCGGGGAGTTCG(-1bp)

C7：TTCAGGTCTTGGTCTCTCGCAGGATG---CTGGAGCCACGTTTATGGCTGCGGGGAGTTCG(-3bp)

C8： CTTGGTCTCTCGCAGGATGTAGTTCAGTCCTGGAGCCACGCTTATGGCTGCGGGGAGTTCG(+7bp)

C9： TTCAGGTCTTGGTCTCTCGCAGGATGTTGCTGGAGCCACGTTTATGGCTGCGGGGAGTTCG(WT)

C10: TTCAGGTCTTGGTCTCTCGCAGGATG---CTGGAGCCACGTTTATGGCTGCGGGGAGTTCG(-3bp)

C11: TTCAGGTCTTGGTCTCTCGCAGGATG---CTGGAGCCACGTTTATGGCTGCGGGGAGTTCG(-3bp)

C12: TTCAGGTCTTGGTCTCTC--------TTGCTGGAGCCACGTTTATGGCTGCGGGGAGTTCG(-8bp)

C13: TTCAGGTCTTGGTCTCT-----------GCTGGAGCCACGTTTATGGCTGCGGGGAGTTCG(-11bp)

C14: CTCTCGCAGGATGTGTATTATGCAGGATGCTGGAGCCACGTTTATGGCTGCGGGGAGTTCG(+13bp)

C15: TTCAGGTCTTGGTCTCTCGCAGGATG---CTGGAGCCACGTTTATGGCTGCGGGGAGTTCG(-3bp)

C16: TTCAGGTCTTGGTCTCTCGCAGGATG---CTGGAGCCACGTTTATGGCTGCGGGGAGTTCG(-3bp)

C17: TTCAGGTCTTGGTCTCTCGCAGGATG---CTGGAGCCACGTTTATGGCTGCGGGGAGTTCG(-3bp)

N-Cas9-C protein：(indel:16,WT:0)

WT：TTCAGGTCTTGGTCTCTCGCAGGATGTTGCTGGAGCCACGTTTATGGCTGCGGGGAGTTCG

B1：TTCAGGTCTTGGTCTCTCGCAGGATG---CTGGAGCCACGTTTATGGCTGCGGGGAGTTCG(-3bp)

B2：AGGTCTTGGTCTCTCGCAGGATGTGCGTGCTGGAGCCACGTTTATGGCTGCGGGGAGTTCG(+3bp)

B3：TTCAGGTCT-------------------GCTGGAGCCACGTTTATGGCTGCGGGGAGTTCG(-19bp)

B4：TTCAGGTCTTGGTCTCTCGCAGGACG-TGCTGGAGCCACGTTTATGGCTGCGGGGAGTTCG(-2bp,+1bp)

B5：TTCAGGTCTTGGTCTCTC---------TGCTGGAGCCACGTTTATGGCTGCGGGGAGTTCG(-9bp)

B6：TTCAGGTCTTGGTCTCTCGCAGGATG---CTGGAGCCACGTTTATGGCTGCGGGGAGTTCG(-3bp)

B7：CAGGTCTTGGTCTCTTTCAGGTCTTGGTGCTGGAGCCACGTTTATGGCTGCGGGGAGTTCG(-5bp,+7bp)

B8: TTCAGGTCTTGGTCTCTCGCAGGATGTTGTTGGAGCCACGTTTATGGCTGCGGGGAGTTCG(-1bp,+1bp)

B9: TTCAGGTCTTGGTCTCTCGCAGGATG---CTGGAGCCACGTTTATGGCTGCGGGGAGTTCG(-3bp)

B10: TTGGTCTCTCGCAGAGACCAAGCCACGTGCTGGAGCCACGTTTATGGCTGCGGGGAGTTCG(+11bp)

B11: TTCAGGTCTTGGTCTCTC---------TGCTGGAGCCACGTTTATGGCTGCGGGGAGTTCG(-9bp)

B12: TTCAGGTCTTGGTCTCTCGCAGGATG---CTGGAGCCACGTTTATGGCTGCGGGGAGTTCG(-3bp)

B13: TTCAGGTCTTGGTCTCTCGCAGGATG-------ACCCACGTTTATGGCTGCGGGGAGTTCG(-7bp)

B14: TTCAGGTCTTGGTCTCTCGCAGGATG---CTGGAGCCACGTTTATGGCTGCGGGGAGTTCG(-3bp)

B15: TTCAGGTCTTGGTCTCTC---------TGCTGGAGCCACGTTTATGGCTGCGGGGAGTTCG(-9bp)

B16: TTCAGGTCTTGGTCTCTCGCAGGATG---CTGGAGCCACGTTTATGGCTGCGGGGAGTTCG(-3bp)

N-Cas9-C mRNA：(indel:9,WT:9)

WT： TCAGGTCTTGGTCTCTCGCAGGATGTTGCTGGAGCCACGTTTATGGCTGCGGGGAGTTCG

m1: TCAGGTCTTGGTCTCTCGCAGGATGTTGCTGGAGCCACGTTTATGGCTGCGGGGAGTTCG(WT)

m2: TCAGGTCTTGGTCTCTCGCAGGATGTTGCTGGAGCCACGTTTATGGCTGCGGGGAGTTCG(WT)

m3: TCAGGTCTTGGTCTCTCGCAG-----------AGCCACGTTTATGGCTGCGGGGAGTTCG(-11bp)

m4: TCAGGTCTTGGTCTCTCGCAGGATGTTGCTGGAGCCACGTTTATGGCTGCGGGGAGTTCG(WT)

m5： TCAGGTCTTGGTCTCTCGCAGGATGTTGCTGGAGCCACGTTTATGGCTGCGGGGAGTTCG(WT)

m6： TCTTGGTCTCTCGCAGGATGAATGTTGTGCTGGAGCCACGTTTATGGCTGCGGGGAGTT(+5bp)

m7： TTCAGGTCTTGGTCTCTCGCAGGATGTTGCTGGAGCCACGTTTATGGCTGCGGGGAGTT(WT)

m8： TTCAGGTCTTGGTCTCTCGCAGGATGTTGCTGGAGCCACGTTTATGGCTGCGGGGAGTT(WT)

m9： TTCAGGTCTTGGTCTCTCGCAG-----------AGCCACGTTTATGGCTGCGGGGAGTT(-11bp)

m10:TTCAGGTCTTGGTCTCTCGCAGGATG---CTGGAGCCACGTTTATGGCTGCGGGGAGTT(-3bp)

m11:CAGGTCTTGGTCTCTCGCAGGA----------GCCACGTTTATGGCTGCGGGGAGTTCG(-10bp)

m12:GATG-------A--------------------------------------CGTCACTGC(-45bp)

m13:TTCAGGTCTTGGTCTCTCGCAGGATGTTGCTGGAGCCACGTTTATGGCTGCGGGGAGTT(WT)

m14:TCTTGGTCTCTCGCAGGATGGAGCCACGTTTA-TGGAGCCACGTTTATGGCTGCGGGGA(-1bp,+10bp)

m15:CAGGTCTTGGTCTCTCGCAGGA----------GCCACGTTTATGGCTGCGGGGAGTTCG(-10bp)

m16:CAGGTCTTGGTCTCTCGCAGGATGTTGCTGGAGCCACGTTTATGGCTGCGGGGAGTTCG(WT)

m17:GTCTTGGTCTCTCGCAGGATGTTGGAATGCTGGAGCCACGTTTATGGCTGCGGGGAGTT(+5bp)

m18:CAGGTCTTGGTCTCTCGCAGGATGTTGCTGGAGCCACGTTTATGGCTGCGGGGAGTTCG(WT)

Figure S8

***gol* target2:**

N-Cas9 protein：(indel:7,WT:13)

WT： TCTAGTGGGCTGGAGTGACGACAGCAGCCTGCGGGTCCAGCGCCGTTCCAGAAATGACA

N1： TCTAGTGGGCTGGAGTGACGACAGCAGCCTGCGGGTCCAGCGCCGTTCCAGAAATGACA(WT)

N2： TCTAGTGGGCTGGAGTGACGACAGCAG------GGTCCAGCGCCGTTCCAGAAATGACA(-6bp)

N3： TCTAGTGGGCTGGAGTGACGACAGCAGCCTGCGGGTCCAGCGCCGTTCCAGAAATGACA(WT)

N4： TCTAGTGGGCTGGAGTGACGACAGCAGC----GGGTCCAGCGCCGTTCCAGAAATGACA(-4bp)

N5： TCTAGTGGGCTGGAGTGACGACAGCAGCCTGCGGGTCCAGCGCCGTTCCAGAAATGACA(WT)

N6： TCTAGTGGG---AATGACA(-43bp)

N7： TCTAGTGGGCTGGAGTGACGACAGCAGCCTGCGGGTCCAGCGCCGTTCCAGAAATGACA(WT)

N8： TCTAGTGGGC-----------CA-GCGCCGT----TCCAGCGCCGTTCCAGAAATGACA(-20bp,+4bp)

N9: TCTAGTGGGCTGGAGTGACGACAGCAGCCTGCGGGTCCAGCGCCGTTCCAGAAATGACA(WT)

N10: TCTAGTGGGCTGGAGTGACGACAGCAGCCTGCGGGTCCAGCGCCGTTCCAGAAATGACA(WT)

N11: TCTAGTGGGCTGGAGTGACGACAGCAGCCTGCGGGTCCAGCGCCGTTCCAGAAATGACA(WT)

N12: TCTAGTGGGCTGGAGTG-----------CTGCGGGTCCAGCGCCGTTCCAGAAATGACA(-11bp)

N13: TCTAGTGGGCTGGAGTGACGACAGCA--CTGCGGGTCCAGCGCCGTTCCAGAAATGACA(-2bp)

N14: TCTAGTGGGCTGGAGTGACGACAGCAG-------GTCCAGCGCCGTTCCAGAAATGACA(-7bp)

N15: TCTAGTGGGCTGGAGTGACGACAGCAGCCTGCGGGTCCAGCGCCGTTCCAGAAATGACA(WT)

N16: TCTAGTGGGCTGGAGTGACGACAGCAGCCTGCGGGTCCAGCGCCGTTCCAGAAATGACA(WT)

N17: TCTAGTGGGCTGGAGTGACGACAGCAGCCTGCGGGTCCAGCGCCGTTCCAGAAATGACA(WT)

N18: TCTAGTGGGCTGGAGTGACGACAGCAGCCTGCGGGTCCAGCGCCGTTCCAGAAATGACA(WT)

N19: TCTAGTGGGCTGGAGTGACGACAGCAGCCTGCGGGTCCAGCGCCGTTCCAGAAATGACA(WT)

N20: TCTAGTGGGCTGGAGTGACGACAGCAGCCTGCGGGTCCAGCGCCGTTCCAGAAATGACA(WT)

C-Cas9 protein：(indel:9,WT:7)

WT： TCTAGTGGGCTGGAGTGACGACAGCAGCCTGCGGGTCCAGCGCCGTTCCAGAAATGACA

C1： TCTAGTGGGCTGGAGTGACGACAGCAGCC---------------GGTCCGTAAATGACA(-17bp,+2bp)

C2： TCTAGTGGGCTGGAGTGACGACAGCAGCC---------------GGTCCGTAAATGACA(-17bp,+2bp)

C3： TCTAGTGGGCTGGAGTG--------------CGGGTCCAGCGCCGTTCCAGAAATGACA(-14bp)

C4： TCTAGTGGGCTGGAGTGACGACAGCAGCCTGCGGGTCCAGCGCCGTTCCAGAAATGACA(WT)

C5： TCTAGTGGGCTGGAGTGAC----------TGCGGGTCCAGCGCCGTTCCAGAAATGACA(-10bp)

C6： TCTAGTGGGCTGGAGTGACGACAGCAGCCTGCGGGTCCAGCGCCGTTCCAGAAATGACA(WT)

C7： TCTAGTGGGCTGGAGTGACGACAGCAGCCTGCGGGTCCAGCGCCGTTCCAGAAATGACA(WT)

C8: TCTAGTGGGCTGGAGTGACGACAGCAGCCTGCGGGTCCAGCGCCGTTCCAGAAATGACA(WT)

C9: TCTAGTGGGCTGGAGTGACGACAGCAG-------GTCCAGCGCCGTTCCAGAAATGACA(-7bp)

C10: TCTAGTGGGCTGGAGTGACGACAGCAGCCTGCGGGTCCAGCGCCGTTCCAGAAATGACA(WT)

C11: TCTAGTGGGCTGGAGTGACGACAGCAGCCTGCGGGTCCAGCGCCGTTCCAGAAATGACA(WT)

C12: TCTAGTGGGCTG-------------------CGGGTCCAGCGCCGTTCCAGAAATGACA(-19bp)

C13: TGACGACAGCAGCAGGGTCGAGGGTCGACTGCGGGTCCAGCGCCGTTCCAGAAATGACA(+12bp)

C14: TCTAGTGGGCTGGAGTGACGACAGCAGCCTGCGGGTCCAGCGCCGTTCCAGAAATGACA(WT)

C15: TCTAGTGGGCTGGAGTGACGAC--------------------------CAGAAATGACA(-26bp)

C16: TCTAG---CCT-----------------CTGCGGGTCCAGCGCCGTTCCAGAAATGACA(-20bp)

N-Cas9-C protein：(indel:12,WT:7)

WT： TCTAGTGGGCTGGAGTGACGACAGCAGCCTGCGGGTCCAGCGCCGTTCCAGAAATGACA

B1： TCTAGTGGGCTGGAGTGACGACAGCAGCCTGCGGGTCCAGCGCCGTTCCAGAAATGACA(WT)

B2： TCTAGTGGGCTGGAGTGACGACAGCAG------GGTCCAGCGCCGTTCCAGAAATGACA(-6bp)

B3： TCTAGTGGGCTGGAGTGACGACAGCAG------GGTCCAGCGCCGTTCCAGAAATGACA(-6bp)

B4： TCTAGTGGGCTGGAGTGACGACAGCAGCCTGCGGGTCCAGCGCCGTTCCAGAAATGACA(WT)

B5： GTGACGACAGCAGCTCTGGGTCTGCAGCTGCGGGTCCAGCGCCGTTCCAGAAATGACAG(+13bp)

B6： TCTAGTGGGCTGGAGTGACAACAGCAGCCTGCGGGTCCAGCGCCGTTCCAGAAATGACA(-1bp,+1bp)

B7： TCTAGTGGGCTGGAGTGACGACAGCAGCCTGCGGGTCCAGCGCCGTTCCAGAAATGACA(WT)

B8： TCTAGTGGGCTGGAGTGACGACAGCAGC----GGGTCCAGCGCCGTTCCAGAAATGACA(-4bp)

B9: TCTAGTGGGCTGGAGTGACGACAGCAGCCTGCGGGTCCAGCGCCGTTCCAGAAATGACA(WT)

B10: GGAGTGACGACAGCATACCGGTATGTACCTGCGGGTCCAGCGCCGTTCCAGAAATGACA(-1bp,+12bp)

B11: TCTAGTGGGCTGGAGTGACGACAGCAGCCTGCGGGTCCAGCGCCGTTCCAGAAATGACA(WT)

B12: TCTAGTGGGCTGGAGTGACGACAGCAG-------GTCCAGCGCCGTTCCAGAAATGACA(-7bp)

B13: TCTAGTGGGC----------------------GGGTCCAGCGCCGTTCCAGAAATGACA(-22bp)

B14: TCTAGTGGGCTGGAGTGACGACAGCAG------GGTCCAGCGCCGTTCCAGAAATGACA(-6bp)

B15: TCTAGTGGGCTGGAGTGACGACAG----------GTCCAGCGCCGTTCCAGAAATGACA(-10bp)

B16: TCTAGTGGGCTGGAGTGACGACAGCAGCCTGCGGGTCCAGCGCCGTTCCAGAAATGACA(WT)

B17: TCTAGTGGGCTGGAGTGACGACAG---------GGTCCAGCGCCGTTCCAGAAATGACA(-9bp)

B18: TCTAGTGGGCTGGAGTGACGACAGCAGCCTGCGGGTCCAGCGCCGTTCCAGAAATGACA(WT)

B19: TCTAGTGGGCTGGAGTGACGACAGC-GCCTGTGGGTCCAGCGCCGTTCCAGAAATGACA(-2bp,+1bp)

N-Cas9-C mRNA：(indel:8,WT:12)

WT：CAGCCTCTAGTGGGCTGGAGTGACGACAGCAGCCTGCGGGTCCAGCGCCGTTCCAGAAATGACA

m1: CAGCTTCTAGTGGGCTGGAGTGACGACAGCAG------GGTCCAGCGCCGTTCCAGAGA(-7bp,+1bp)

m2： CAGCTTCTAGTGGGCTGGAGTGACGACAGCAG------GGTCCAGCGCCGTTCCAGAGA(-7bp,+1bp)

m3： TCTAGTGGGCTGGAGTGGCGACAGCAGCCTGCGGGTCCAGCGCCGTTCCAGAAATGACA(-1bp,+1bp)

m4： TCTAGTGGGCTGGAGTGACGACAGCAGC----GGGTCCAGCGCCGTTCCAGAAATGACA(-4bp)

m5： TCTAGTGGGCTGGAGTGACGACAGCAGCCTGCGGGTCCAGCGCCGTTCCAGAAATGACA(WT)

m6： TCTAGTGGGCTGGAGTGACGACAGCAGCCTGCGGGTCCAGCGCCGTTCCAGAAATGACA(WT)

m7： TCTAGTGGGCTGGAGTGACGTCAGCA------GGGTCCAGCGCCGTTCCAGAAATGACA(-6bp)

m8： TCTAGTGGGCTGGAGTGACGACAGCAGCCTGCGGGTCCAGCGCCGTTCCAGAAATGACA(WT)

m9： TCTAGTGGGCTGGAGT--------------------CCAGCGCCGTTCCAGAAATGACA(-20bp)

m10: TCTAGTGGGCTGGAGTGACGACAGCAGC----GGGTCCAGCGCCGTTCCAGAAATGACA(-4bp)

m11: TCTAGTGGGCTGGAGTGACGACAGCAGCCTGCGGGTCCAGCGCCGTTCCAGAAATGACA(WT)

m12: TCTAGTGGGCTGGAGTGACGACAGCAGCCTGCGGGTCCAGCGCCGTTCCAGAAATGACA(WT)

m13: TCTAGTGGGCTGGAGTGACGACAGCAGCCTGCGGGTCCAGCGCCGTTCCAGAAATGACA(WT)

m14: TCTAGTGGGCTGGAGTGACGACAGCAGCCTGCGGGTCCAGCGCCGTTCCAGAAATGACA(WT)

m15: TCTAGTGGGCTGGAGTGACGACAGCAGCCTGCGGGTCCAGCGCCGTTCCAGAAATGACA(WT)

m16: TCTAGTGGGCTGGAGTGACGACAGCAGCCTGCGGGTCCAGCGCCGTTCCAGAAATGACA(WT)

m17: TCTAGTGGGCTGGAGTGACGACAGCAGCCTGCGGGTCCAGCGCCGTTCCAGAAATGACA(WT)

m18: TCTAGTGGGCTGGAGTGACGACAGCAG-------GTCCAGCGCCGTTCCAGAAATGACA(-7bp)

m19: TCTAGTGGGCTGGAGTGACGACAGCAGCCTGCGGGTCCAGCGCCGTTCCAGAAATGACA(WT)

m20: TCTAGTGGGCTGGAGTGACGACAGCAGCCTGCGGGTCCAGCGCCGTTCCAGAAATGACA(WT)

Table S1. Target sites and target region primers for *tyr* gene and *gol* gene.

	Target site	Forward primer	Reverse primer
tyr1	CCCCAGAAGTCCTCCAGTCC	GCGTCTCACTCTCCTCGACTCTTC	GTAGTTTCCGGCGCACTGGCAG
tyr2	GGCTGACGGACTACGAGACC	AATGTCGTTCACTCTGCTGTT	TGGCTTTAAAGTGGACGCTG
gol1	GGTCTCTCGCAGGATGTTGC	AGGTCATCAGCGAGCGTAAG	CACTGACGGATCTCTGCACT
gol1'		TGCAGTTTTTCTTGGTGTTCTTTGA	TTTCTCTCATATATCTACCCGATAG
gol2	GGAGTGACGACAGCAGCCTG	TGTGTGTGCAGGTATGATGG	CAGGCTGAGCGATAGATGTG

* *gol1'* primers used for the second and third experiment and multiplex injection experiment.
